# Sensing their plasma membrane curvature allows migrating cells to circumvent obstacles

**DOI:** 10.1038/s41467-023-41173-1

**Published:** 2023-09-13

**Authors:** Ewa Sitarska, Silvia Dias Almeida, Marianne Sandvold Beckwith, Julian Stopp, Jakub Czuchnowski, Marc Siggel, Rita Roessner, Aline Tschanz, Christer Ejsing, Yannick Schwab, Jan Kosinski, Michael Sixt, Anna Kreshuk, Anna Erzberger, Alba Diz-Muñoz

**Affiliations:** 1 Cell Biology and Biophysics Unit, European Molecular Biology Laboratory, 69117 Heidelberg, Germany; 2 Collaboration for joint PhD degree between EMBL and Heidelberg University, Faculty of Biosciences, EMBL and Heidelberg University, Heidelberg, Germany; 3 Institute of Science and Technology Austria, 3400 Klosterneuburg, Austria; 4 EMBL Hamburg, European Molecular Biology Laboratory, 22607 Hamburg, Germany; 5 Centre for Structural Systems Biology, 22607 Hamburg, Germany; 6 Department of Biochemistry and Molecular Biology, Villum Center for Bioanalytical Sciences, University of Southern Denmark, Campusvej 55, 5230 Odense, Denmark; 7 Structural and Computational Biology Unit, European Molecular Biology Laboratory, 69117 Heidelberg, Germany; 8 Present Address: Division of Medical Image Computing, German Cancer Research Center (DKFZ), 69120 Heidelberg, Germany

**Keywords:** Membrane biophysics, Cell migration

## Abstract

To navigate through diverse tissues, migrating cells must balance persistent self-propelled motion with adaptive behaviors to circumvent obstacles. We identify a curvature-sensing mechanism underlying obstacle evasion in immune-like cells. Specifically, we propose that actin polymerization at the advancing edge of migrating cells is inhibited by the curvature-sensitive BAR domain protein Snx33 in regions with inward plasma membrane curvature. The genetic perturbation of this machinery reduces the cells’ capacity to evade obstructions combined with faster and more persistent cell migration in obstacle-free environments. Our results show how cells can read out their surface topography and utilize actin and plasma membrane biophysics to interpret their environment, allowing them to adaptively decide if they should move ahead or turn away. On the basis of our findings, we propose that the natural diversity of BAR domain proteins may allow cells to tune their curvature sensing machinery to match the shape characteristics in their environment.

## Abstract (web summary)

Motile cells must navigate complex environments. Here the authors use state-of-the-art imaging, coarse-grained MD simulations and experimental biophysics to show that cells sense their plasma membrane curvature to circumvent obstacles.

## Introduction

Cell migration drives many developmental, physiological, and pathological processes. While the mechanisms underlying propulsion are largely known, it remains unclear how cells steer their movement to navigate complex and dynamic environments^1^ and circumvent obstacles within tissues^2,3^. These adaptive behaviors are particularly important for fast and dynamic cell types, such as immune cells and disseminating tumor cells. Switching between actin-based protrusions and blebbing has been shown to aid cell steering in development^4^, but cells often display only actin-rich protrusions such as lamellipodia and ruffles. These need to be long-lived enough for cells to explore and then persistently move through their surroundings, while ‘unstable’ enough to allow them to adapt and change direction when confronted with an obstacle^5,6^.

During migration, the outmost boundary of the cell—the plasma membrane—is deformed by the changing microenvironment. However, it remains unclear whether membrane curvature encodes information that cells use to choose their migration path^7,8^, or if membrane topography is only a side-effect of the forces acting at the cell surface. Many migrating cells indeed express a variety of curvature-sensing proteins, which can directly interact with both the plasma membrane and the subjacent actin cytoskeleton^9^. In particular, the family of BAR domain proteins could facilitate membrane curvature sensing. These proteins form crescent-shaped membrane-binding dimers that can sense and generate curvature^9–11^, and are known to interact with regulators of the actin cytoskeleton such as the Arp2/3 complex, formins, or Rho GTPases through several auxiliary domains^9,12^. In fact, changes in surface topography induce recruitment of some cytosolic BAR domain proteins to regions of highly positive (inward) curvature^13^, and can trigger the formation of actin structures and endocytosis hotspots^14,15^. Here, we show that the curvature-sensing BAR domain protein Snx33 inhibits actin polymerization and rearranges the localization of WAVE2 to limit the persistence of the leading edge and steer migration. By this mechanism Snx33 allows cells to adaptively decide if they should move ahead or turn away.

## Results

### Snx33 is preferentially excluded from outward-curved membrane regions at the leading edge of migrating cells

**Fig. 1:**
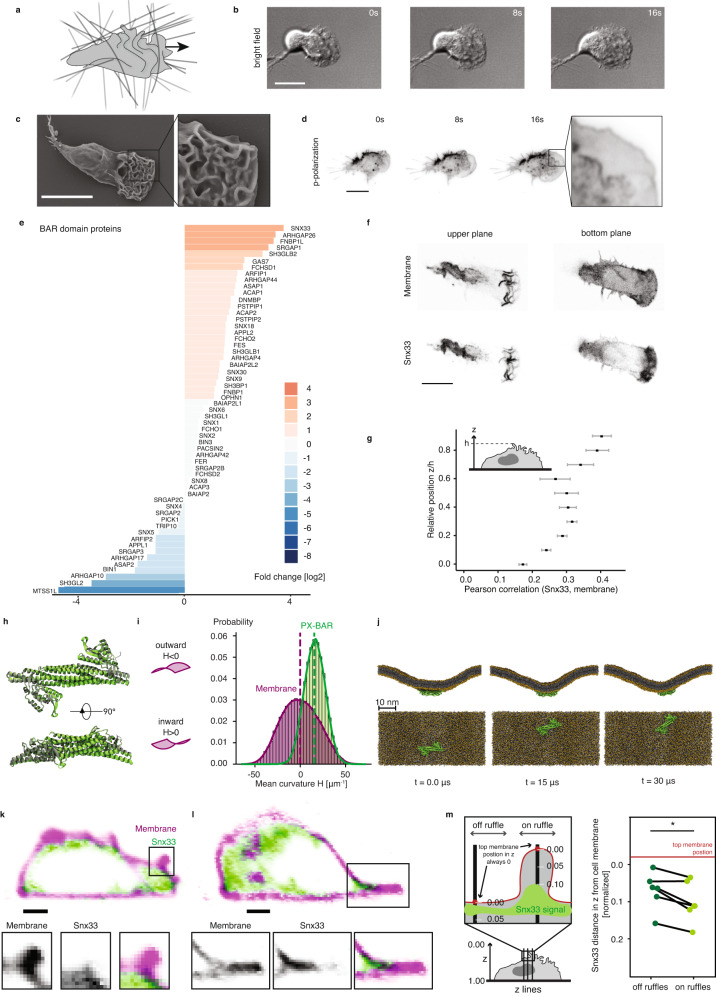
Curvature patterning in the lamellipodium and the curvature-sensitive protein Snx33. **a** The leading edge of migrating cells is characterized by intricate curvature patterns. The arrow indicates the direction of cell movement. Lines depict a complex 3D environment. **b** Time-lapse bright-field imaging shows migrating immune-like differentiated HL-60 cells. **c** Scanning electron microscopy (SEM) image of a wild-type cell with zoom-in at the leading edge. *n* = 175. **d** Time-lapse p-polarization of p-TIRFM imaging reveals dynamic membrane waves at the leading edge. **e** Up-regulated (orange) and down-regulated (blue) BAR domain genes between differentiated (migratory) and undifferentiated (non-migratory) HL-60 cells. **f** Fluorescently tagged Snx33 (eGFP-Snx33) and membrane marker (CAAX-mcherry) in upper (close-to-the-cell-top) and lower (near-surface plane) *z*-planes in a cell. **g** Pearson correlation coefficient of fluorescently tagged Snx33 and membrane marker at different heights acquired by confocal microscopy. *n* = 10. Error bars denote the standard error of the mean. **h** Structure of the Snx33 PX-BAR domain as modeled with AlphaFold-multimer (green) compared with the incomplete crystal structure (PDB ID: 4AKV; gray). **i** Probability histogram of the mean curvature (H) sampled at the center of mass of PX-BAR (green, mean curvature_PX-BAR_ = 16 μm^−1^) and at a random lipid phosphate position (yellow). Kernel density estimates to smooth the distributions are shown. The mean values are indicated as dashed vertical lines. **j** Top and side views from snapshots of the coarse-grained simulation of a buckled membrane with the PX-BAR domain. Protein backbone beads (green), phosphate beads (yellow spheres), and lipid tails (gray sticks) are shown. Water and ions are omitted for clarity. A top and side view are shown. Time points are indicated for the respective frames. **k**, **l** Cross-sections (*xz*) of migrating cells using lattice-light-sheet microscopy visualizing eGFP-Snx33 and CAAX-mcherry with a zoom-in on a membrane ruffle (**k**), and on a lamellipodium (**l**). *n* = 6. **m** Quantification of the average *z*-position of Snx33 relative to the plasma membrane on- and off-ruffles (*p*_TopMembrane_ = 0.01635, *t* = −3.5524, df = 5, paired, two-sided). *n* = 6 cells which data were obtained from two-color 3D movies. Each point represents the average for 1 cell. Scale bars = 10 μm. *p* < 0.05 (*).

During the differentiation process immune-like HL-60 cells undergo substantial changes in gene expression, initiate rapid migration, and acquire a complex membrane topography, resembling what occurs in the bone marrow in vivo^16^ (Supplementary Fig. 1a). The terminally differentiated, motile cells (dHL-60 cells) display both actin-rich lamellipodia and membrane ruffles at the leading edge (Fig. 1a, b). To analyze membrane topography in more detail, we used scanning electron microscopy (SEM), for ultrastructural detail of natively fixed cell membranes, and polarized total internal reflection fluorescence microscopy (p-TIRFM), which enables probing membrane curvature dynamics in live cells. We observed curved membrane patterns, as seen by SEM in the upper plasma membrane (Fig. 1c), that are very dynamic, as visualized by p-TIRFM in the basal plasma membrane (Fig. 1d, Supplementary Fig. 1b–d, and Supplementary Video 1).

These curved membrane patterns likely constitute binding sites for curvature-sensitive proteins, such as those from the BAR domain family. To identify candidates from this large family that could sense indenting obstacles and thus be relevant for adaptive behaviors during cell migration, we measured their expression profile before and after HL-60 differentiation to a migratory state, i.e., in a curvature-poor (blasts are uniformly round) versus a curvature-rich state (Supplementary Fig. 1a). The expression of the BAR domain protein Snx33 increased 16-fold during this differentiation process (Fig. 1e). We imaged its subcellular localization by confocal microscopy after fluorescent tagging (eGFP-Snx33) and found that Snx33 is enriched throughout membrane ruffles (Fig. 1f, g), the highly curved structures at the leading edge of dHL-60 cells. Based on the similarity within its protein family, Snx33 is predicted to bind shallow curvatures in comparison to many other BAR domain proteins from the BAR subgroup^9^. The membrane-binding domain of Snx33 (PX-BAR) contains a positively charged patch on the convex surface, which strongly suggests an inward curvature-dependent membrane-binding on this surface (Supplementary Fig. 2a–d). To assess its curvature-sensing properties in more detail, we performed long coarse-grained molecular dynamics (MD) simulations of the PX-BAR domain (Fig. 1h–j) on buckled membranes with plasma membrane-derived lipid composition (Table S1; see Methods for details). This computational assay has been used extensively to characterize curvature-sensing properties of a wide variety of proteins^17,18^, including other BAR domains^19^. During the 30 µs long simulation, the PX-BAR domain of Snx33 remained tightly bound to the membrane and showed a strong preference for membrane regions with inward local curvature (Fig. 1i, j, Supplementary Fig. 2e, f). Snx33 binds the plasma membrane with a surface of basic residues such as lysine and arginine that mediate electrostatic interactions (Supplementary Figs. 2a–d, 3a, b, 4a–g, see Supplemental Discussion for details), which is common for plasma membrane binding^20^. To further test the curvature sensitivity of Snx33 in cells, we imaged its subcellular localization by lattice light sheet microscopy and, supporting our MD simulations, we found that Snx33 is excluded from the ruffle tips and lamellipodium edge, both structures with highly negative (outward) curvature (Fig. 1k–m, Supplementary Fig. 5, and Supplementary Video 2, see Methods for details). To corroborate the tip exclusion of Snx33, we compared its localization to that of IRSp53 (also known as BAIAP2), a canonical outward curvature-binding protein^21^. As previously reported, in neutrophil-like cells IRSp53 accumulates at the tip of the advancing front and retracting fibers^22,23^, known outward-curved structures (Supplementary Fig. 6a–c and Supplementary Video 3). Altogether, we show that the BAR domain-containing protein Snx33 has a strong preference for membrane regions with inward local curvature and is enriched at the leading edge but excluded from outward-curved membrane structures.

### Snx33 controls leading edge growth and modulates plasma membrane tension

**Fig. 2:**
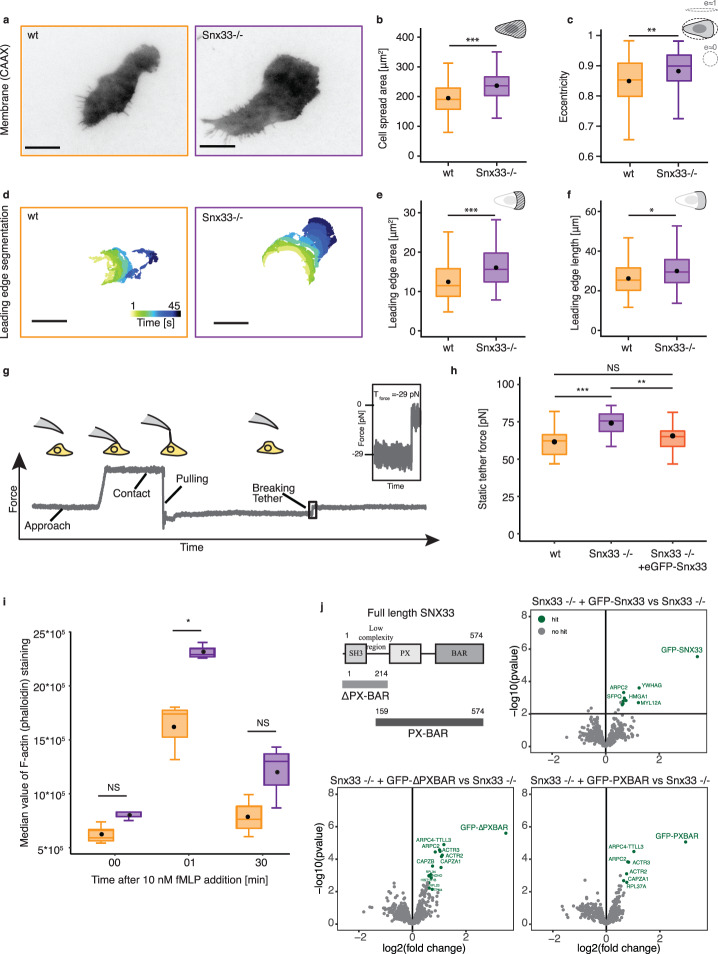
Snx33 knockout cells display altered cell and leading edge morphology due to an increase in actin polymerization. **a** TIRFM images of a wild-type and a Snx33-/- cell. **b** Cell spread area (*p* = 1.505e-7, Mann–Whitney-*U*-test, two-sided) and **c** eccentricity differ between wild-type and Snx33-/- cells during movement (*p* = 0.004989, Mann–Whitney-*U*-test, two-sided). **d** Leading edge segmentation of a wild-type and a Snx33-/- cell. Time frames (5 s) are color-coded. **e** Leading edge area (*p* = 1.634e-5, Mann–Whitney-*U*-Test, two-sided) and **f** length (*p* = 0.2111, Mann–Whitney-*U*-test, two-sided). *n* = 82 (wt), *n* = 78 (Snx33-/-). **g** Schematic of static tether pulling experiments. **h** Mean static tether force of wt (*n* = 24), Snx33-/- (*n* = 26) and Snx33-/- with overexpressed eGFP-Snx33 (*n* = 25) from 3 independent experiments (*p*_wt vs. Snx33-/-_ = 7.883e-6, t = −5.0144, df = 47.376; *p*_wt vs. Snx33-/- + eGFP-Snx33_ = 0.2141, *t* = −1.2601, df = 45.515; *p*_Snx33-/- vs. Snx33-/- + eGFP-Snx33_ = 0.2141, *t* = −1.2601, df = 45.515, two-sided). **i** Median value of phalloidin fluorescence intensity in fixed wt and Snx33-/- dHL-60 cells quantified by flow cytometry. Data from 3 independent experiments (*p*_t00_ = 0.184, Mann–Whitney-*U*-test, two-sided; *p*_t01_ = 0.0373, *t* = −4.26, df = 2.375, two-sided; *p*_t30_ = 0.1298, *t* = −1.9728, df = 3.5035, two-sided). **j** Schematic of full-length Snx33 with its domains and two Snx33 truncations (ΔPXBAR and PXBAR). Volcano plots from co-immunoprecipitation experiments comparing Snx33-/- with Snx33-/- + GFP-Snx33, Snx33-/- + GFP-Snx33ΔPXBAR and Snx33-/- + GFP-Snx33PXBAR. Enriched hits (limma *p*-value ≦ 0.01, fold-change ≧ 50%) are shown green, and the rest gray. Scale bars = 10 μm. *p* < 0.001 (***), *p* < 0.01 (**), *p* < 0.05 (*). Box-plots: lower and upper hinges correspond to the 25th and 75th percentile. The upper whisker extends from the hinge to the largest value, but no further than 1.5*IQR. The lower whisker extends from the hinge to the smallest value, but no lower than 1.5*IQR of the hinge. Data beyond the whiskers: black dots. Black line: median. Black dot: mean.

To study the role of Snx33 in adaptive migration, we generated a Snx33 knockout (Snx33-/-) cell line using CRISPR/Cas9 genome editing (Supplementary Fig. 7a, b). Propulsion in immune-like cells depends on the active polymerization of actin at the leading edge of the cell^24,25^. Given that cell shape reflects changes in motion-driving actin-rich protrusions^26^, we performed a quantitative and unbiased comparison of selected cell morphometric parameters, i.e., cell spreading, cell eccentricity, and leading-edge characteristics. As immune cells radically change their morphology in short periods of time, we averaged time-lapse movies to capture their dynamics. To this end, we trained and used machine-learning-based algorithms in ilastik^27^ to analyze movies of cells imaged by total internal reflection fluorescence microscopy (TIRFM) (see Methods for details). Snx33-/- cells spread to a larger extent, and showed a more elongated morphology and bigger leading edge (Fig. 2a–f, Supplementary Fig. 8a–e, and Supplementary Videos 4 and 5), independent of adhesion to the substrate (Supplementary Fig. 8f–i). Moreover, the increase in spread area and leading edge area could be rescued by expressing fluorescently tagged Snx33 proving the specificity of the observed phenotypes (Supplementary Fig. 8b, c). Altogether, these results indicate that Snx33-/- cells have a more stable leading edge during migration.

Leading edge growth and increased cell spreading during persistent migration is known to increase plasma membrane tension^28,29^. To test if the more stable leading edge induced by the loss of Snx33 leads to higher membrane tension, we measured it in the leading edge by static tether pulling using atomic force spectroscopy, where a plasma membrane tether is held with a constant length until it breaks (Fig. 2g). We found that the static tether force was significantly increased in Snx33-/- dHL-60 cells (from 61.58 to 75.25 pN, Fig. 2h), which corresponds to an almost 50% increase in apparent membrane tension (from 177.87 to 265.62 μN/m; see Methods for details). Moreover, the increase in membrane tension could be rescued by stably expressing fluorescently tagged Snx33, excluding Snx33-independent functions as the origin of this phenotype (Fig. 2h). Notably, overexpression of Snx33-GFP on the wild-type background did not decrease membrane tension, suggesting that a gain of function is not sufficient to alter the leading edge or its effects on membrane mechanics (Supplementary Fig. 9a). Last, these phenotypes were not a consequence of defective differentiation, as the neutrophil differentiation marker CD11b was unperturbed in Snx33-/- cells, nor of potential off-target effects caused by cell line generation by CRISPR/Cas9 technology (Supplementary Fig. 9b, c). Altogether, these results confirm that Snx33-/- cells have a more stable leading edge showing all the characteristics expected for persistent migration.

### Snx33 regulates actin polymerization

Our findings suggest that the curvature-sensing protein Snx33 negatively regulates actin polymerization and thereby limits leading edge size. Thus, we quantified the amount of filamentous actin (F-actin) by phalloidin staining upon chemoattractant (fMLP) addition by flow cytometry and observed a significant increase in the total amounts of F-actin in Snx33-/- cells when compared with their wild-type counterparts (Fig. 2i). This suggests that Snx33 plays an inhibitory role on actin polymerization, particularly during the initial burst after stimulation. But how does Snx33 inhibit actin polymerization? Some BAR domain proteins remodel actin by binding to it directly or by the recruitment of Nucleation Promoting Factors (NPFs) that activate the Actin Related Protein 2/3 (Arp2/3) complex^9,30–34^. To understand how Snx33 inhibits actin polymerization, we coimmunoprecipitated full-length GFP-Snx33 and its binding partners in the knockout background followed by mass spectrometry. As it is common among BAR domain proteins to have intramolecular autoinhibitory interactions, we also performed coimmunoprecipitation of two truncations of Snx33 containing or missing the PX-BAR domain, but altogether spanning the full-length protein (Fig. 2j). Several members of the Arp2/3 complex as well as two subunits of capping proteins coimmunoprecipitated with Snx33 (limma *p*-value ≦ 0.01, fold-change ≥ 50%) (Fig. 2j). In particular, we identified positive fold-changes for ARPC2, ARPC4-TTLL3, ACTR2, ACTR3, CAPZA1 and CAPZB1 in the full-length GFP-Snx33 or its truncations, suggesting that Snx33 binds to the Arp2/3 complex and to capping proteins. The Arp2/3 complex is the actin nucleator responsible for lamellipodia and ruffles formation at the leading edge of neutrophil cells^35^, while capping proteins terminate the growth of actin filaments by binding to them, and potentiate their branching by the Arp2/3 complex^36–39^. Interestingly, we could observe that some Arp2/3 components and capping protein subunits were substantially enriched with either domain compared to the full-length protein (Supplementary Fig. 10a), suggesting that diverse domains of Snx33 bind them with different affinities, possibly because of autoinhibitory interactions that may occlude binding sites or affect membrane binding, as reported for other BAR domain proteins^40–42^. In summary, Snx33 negatively regulates actin polymerization, likely through the regulation of the Arp2/3 complex and/or capping proteins.

### Snx33 affects WAVE2 localization and ruffle morphology

**Fig. 3:**
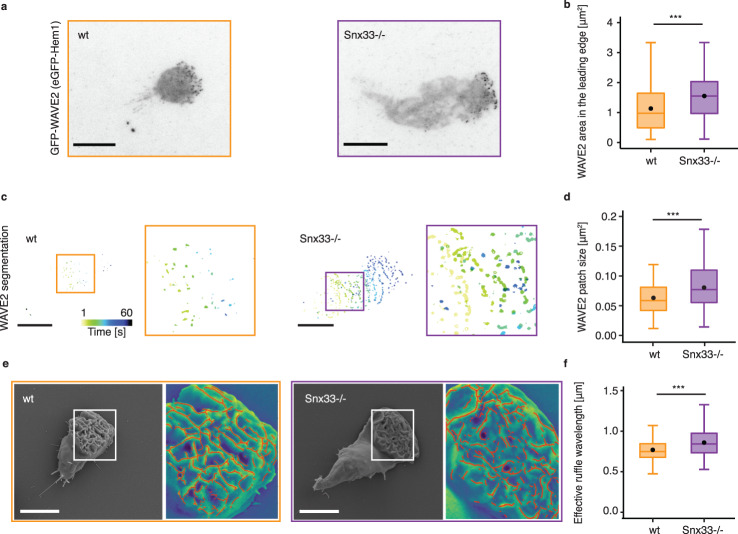
WAVE2 pattern width and ruffle wavelength are increased in Snx33 knockout cells. **a** TIRFM images of a wild-type and Snx33-/- cell show the distribution of WAVE2, the only actin nucleator promoting factor upstream of the Arp2/3 complex in neutrophils. **b** The total area occupied by WAVE2 patches in the leading edge increases upon loss of Snx33 (*p* = 0.000408, Mann–Whitney-*U*-test, two-sided). *n* = 82 (wt), *n* = 78 (Snx33-/-). **c** Segmentation of dynamic WAVE2 patches in a wild-type and Snx33-/- cell. Time frames (5 s) are color-coded. **d** The size of WAVE2 patches increases upon loss of Snx33 (*p* = 0.000464, Mann–Whitney-*U*-test, two-sided). *n* = 82 (wt), *n* = 78 (Snx33-/-). **e** SEM images with ruffle segmentations (red) show the leading edges of a wild-type and Snx33-/- cells from the 50^th^ percentile of the distribution. **f** The effective ruffle wavelength increases upon loss of Snx33 (*p* = 3.171e-7, Mann–Whitney-*U*-test, two-sided). *n* = 175 (wt), *n* = 170 (Snx33-/-). Statistics: *t*-test or non-parametric Mann–Whitney-*U*-test. Scale bars = 10 μm. *p* < 0.001 (***), *p* < 0.01 (**), *p* < 0.05 (*). Box-plots: the lower and upper hinges correspond to the 25th and 75th percentile. The upper whisker extends from the hinge to the largest value, but no further than 1.5*IQR. The lower whisker extends from the hinge to the smallest value, but no lower than 1.5*IQR of the hinge. Data beyond the whiskers: black dots. Black line: median. Black dot: mean.

WAVE2 is the only known actin NPF upstream of the Arp2/3 complex in neutrophils^24,35^. Thus, to further characterize the leading edge of Snx33-/- cells we imaged a component of the WAVE2 complex (Hem-1) during migration by TIRFM (hereafter referred to as WAVE2) (Fig. 3a and Supplementary Video 6 and 7). WAVE2 earned its name because it localizes in waves on the basal membrane during cell migration^24^. To generate such patterns, WAVE2 waves deposit an inhibitor in their wake that transiently inhibits WAVE2 recruitment^24^. Interestingly, polymerized actin is a key component of this inhibitory feedback but what regulates WAVE2 binding to the membrane and determines its patch morphology remains poorly understood^43^. Thus, we quantified WAVE2 pattern characteristics using machine-learning-based segmentation. Strikingly, Snx33-/- cells showed a 40% increase in the WAVE2 area and the size of its patches at the plasma membrane (Fig. 3b–d). To determine whether this is due to a difference in the expression levels of WAVE2 components or due to increased membrane binding, we performed RNAseq. We observed none or a very minor difference in expression in Snx33-/- cells when compared with their control counterparts (Supplementary Fig. 10b), suggesting that Snx33 affects the localization of the WAVE2 complex rather than its expression. This is particularly interesting as, to date, only very drastic WAVE2 patch phenotypes have been reported (complete abrogation or increase in number), which lead to a severely disrupted capacity to migrate^44,45^. We next sought to test the functional relevance of the change in WAVE2 patch morphology for leading-edge morphology. To this end, we quantified the effective ruffle wavelength from SEM images and observed a significantly less tight arrangement of ruffles for Snx33-/- cells when compared with their wild-type counterparts (Fig. 3e, f, Supplementary Fig. 8j, Supplementary Fig. 11a). Last, to determine whether the increase in effective ruffle wavelength is merely the consequence of increase membrane tension in Snx33-/- cells, we performed SEM on PLD2 knock-down (KD) cells, which also display increased membrane tension^44^. Notably, PLD2 KD cells showed no differences in effective ruffle wavelength compared to their Nonsense counterparts (Supplementary Fig. 11b, c). These findings identify Snx33 as a link between membrane shape and the actin polymerization factors that modulate it.

### Snx33 enables curvature-dependent object evasion

**Fig. 4:**
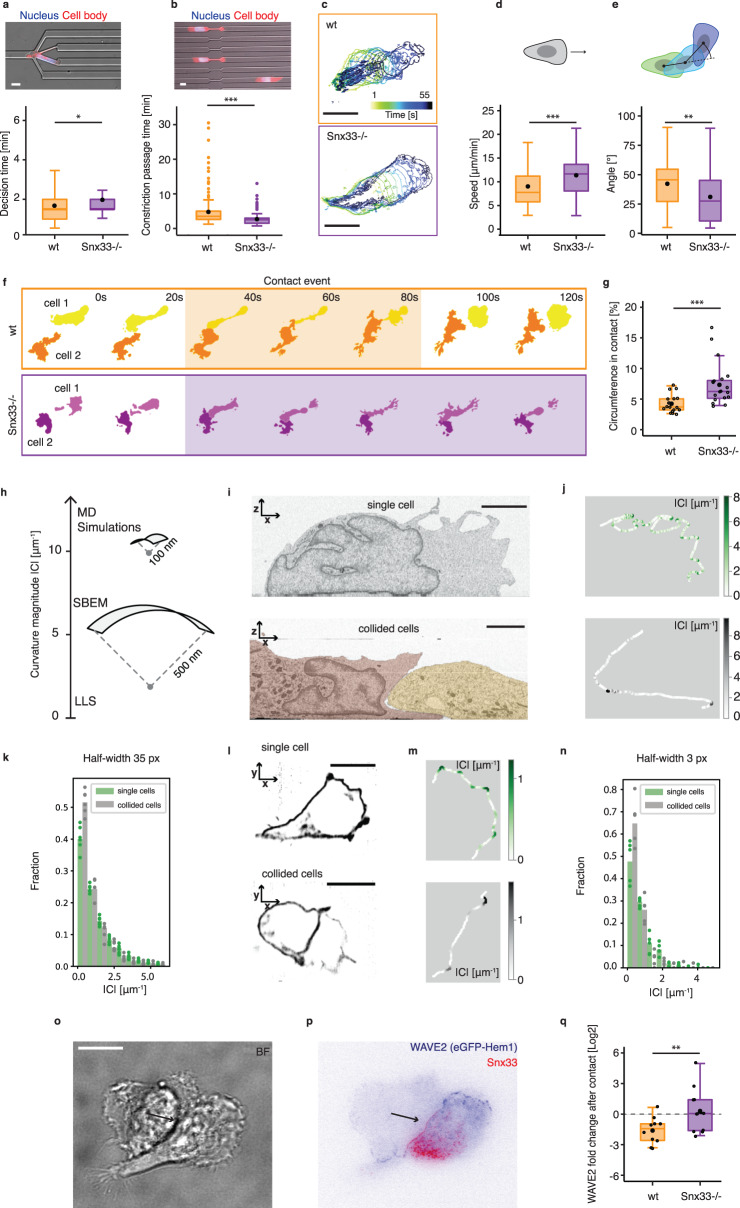
Snx33 steers cell movement in single-cell 2D and 3D migration by inhibiting the WAVE2 complex. **a** Decision channel passage time (*n*_wt_ = 159, mean = 1.67 min; *n*_Snx33-/-_ = 9; mean = 1.96 min) in cells (*p* = 0.02, Mann–Whitney-*U*-*t*est, two-sided). **b** Constriction passage time (*n*_wt_ = 234, *n*_Snx33-/-_ = 158) in cells (*p* = 2.2e-16, Mann–Whitney-*U*-test, two-sided). **c** Cell body displacement over time in wt and Snx33-/- cells. Time frames (5 s) are color-coded. **d** Cell speed in wt and Snx33-/- cells (*p* = 7.661e-5, Mann–Whitney-*U*-test, two-sided) **e** Distribution of angles at which cells turn during migration (*p* = 0.0007583, Mann–Whitney-*U*-Test, two-sided). *N*_wt_ = 82, *n*_Snx33-/-_ = 78. Data from 3 independent biological replicates. **f** Segmentation of a contact event between two wt or Snx33-/- dHL-60 cells. Shade highlights the contact duration. **g** Percentage of cell circumference in contact with another cell in wt (*n* = 18) and Snx33-/- (*n* = 20) dHL-60 cells (*p* = 0.0001704, Mann–Whitney-*U*-test, two-sided). **h** Visualization of curvature magnitudes measured using various techniques. **i** Cross-section of a single and collided migrating dHL-60 cell by SBEM imaging. *N*_single_ = 6, *n*_collided_ = 6. **j** Visualization of the absolute curvature value in the leading edge of single and collided migrating cell (from **i**). **k** Histogram showing the distribution of absolute curvature values in the leading edge of single and collided cells (*n*_single_ = 6, *n*_collided_ = 6). Data points denote an average value per cell. **l** *xy* plane of a membrane marker (CAAX) of a single and collided migrating dHL-60 cells by lattice-light-sheet. **m** Visualization of the absolute curvature value in the leading edge of single and collided migrating cell (from **l**). **n** Histogram showing absolute curvature in the leading edge of single and collided cells from lattice light sheet images (*n*_single_ = 5, *n*_collided_ = 5). Data points denote an average per cell. **o** Bright-field and **p** TIRFM imaging of cell–cell contact in wt dHL-60 cells with fluorescently tagged Snx33 and Hem1. Arrow points towards cell–cell contact. *n* = 3. **q** WAVE2 fold-change after cell–cell contact in wt (*n* = 9) and Snx33-/- (*n* = 10) dHL-60 cells (*p* = 0.01816, *t* = −2.5877, df = 18.789, two-sided). Scale bars = 10 μm. *p* < 0.001 (***), *p* < 0.01 (**), *p* < 0.05 (*). Box-plots: the lower and upper hinges correspond to the 25th and 75th percentile. The upper whisker extends from the hinge to the largest value, but no further than 1.5*IQR. The lower whisker extends from the hinge to the smallest value, but no lower than 1.5*IQR of the hinge. Data beyond the whiskers: black dots. Black line: median. Black dot: mean.

Our data thus far reveal that the membrane curvature-binding protein Snx33 regulates actin polymerization, leading-edge morphology, and stability. Leading edge patterning may facilitate adaptive motility by reducing persistence and promoting random evasive maneuvers. But the particular form of Snx33-mediated membrane-actin coupling suggests an additional, more direct effect, in which propulsion is reduced specifically where the presence of an obstacle in the path of the cell is likely, i.e., where external indentations generate an increased fraction of regions with inward curvature. Thus, Snx33 could facilitate cell steering, because membrane deformations generated by collisions would locally down-regulate propulsion and thereby reorient the leading edge. To test this hypothesis, we first positioned the cells in microfluidic devices^46,47^ where they migrated through channels and encountered obstacles in the form of differently sized pores. Snx33-/- dHL60 cells required almost 20% more time than their wild-type counterparts to navigate these decision-points and to find the path of least resistance (Fig. 4a, Supplementary Fig. 12a, b, and Supplementary Video 8). As the nucleus has been shown to function as a mechanical guide along the path of least resistance^46^, we measured nuclear stiffness by atomic force indentation and ruled out that nuclear mechanics was affected in Snx33-/- cells (Supplementary Fig. 12c, d), in agreement with their retained ability to read out pore size (Supplementary Fig. 12b). However, in decision-free channels, Snx33-/- cells migrated 80% faster than their wild-type counterparts, including during passage through a single constriction (Fig. 4b, Supplementary Fig. 12e, and Supplementary Video 9). Next, we quantified unconfined migration on 2D planar substrates with a homogeneous chemoattractant, promoting cell motility without requiring the cells to adapt to any obstacle (Fig. 4c, see Methods for details). As in decision-free channels, Snx33-/- cells migrated faster than wild-type cells (Fig. 4d). Moreover, while the motility of Snx33-/- cells was more persistent, wild-type cells were more prone to perform large spontaneous turns (Fig. 4e). Thus, the loss of Snx33 appears to render cells less prone to spontaneous turning and less effective in circumnavigating an object while displaying more persistent migration in decision-free environments. These migratory phenotypes are consistent with the increase in leading edge size and plasma membrane tension we have observed in Snx33-/- cells (Fig. 2d–h). Together, these observations suggest that Snx33-/- cells have improved propulsion but lack the ability to adapt when faced with an obstacle.

To gain further insights into the object-evasion response of Snx33-deficient cells, we devised a reductionistic assay to test contact inhibition of locomotion (CIL), a common phenomenon in many cell types, including dHL-60s, where cells stop moving in a particular direction when they come into contact with another cell or an object^24,48^. To test if CIL as a response to cellular obstacles is controlled by Snx33, we seeded a higher density of dHL-60 cells and imaged their 2D migration in the presence of cell–cell interactions by TIRFM. Snx33-/- cells formed larger cell–cell contacts compared to wild-type cells (Fig. 4f, g), in agreement with their increased decision time when faced with an inert obstacle (Fig. 4a). Our computational results together with the observed localization of Snx33 (Fig. 1h–m) suggest preferential binding to inward-curved regions. To assess what changes in plasma membrane geometry, occur upon collisions between cells, we quantified the curvature differences at the leading edge of freely moving cells and of pairs after collision. We performed serial block-face scanning electron microscopy (SBEM), manually segmented the plasma membrane, and quantified the curvatures at free and contacting areas in the front of colliding and single cells in the *xz* plane (Fig. 4h–k). Additionally, to rule out potential fixation artefacts, we analyzed leading edges in freely migrating and colliding live cells using a membrane marker (CAAX) and lattice light sheet (LLS) imaging in the *xy* plane (Fig. 4l–n and Supplementary Fig. 13f–i). In both datasets, we observed a change in the curvature distribution at the front between single and collided cells. While single cells displayed a broader distribution biased towards outward curvatures, the contact surfaces were characterized by narrower distributions centered at zero, in line with an overall flattening at the contact site. Cell–cell contacts suppressed both positive and negative curvatures (Supplementary Fig. 14a–d).

To further dissect the molecular role of Snx33 in CIL, we simultaneously imaged the neutrophil WAVE2 complex (using Hem1) and Snx33 at the leading edge of live cells. While both proteins largely colocalized in most parts of the cell, they were anticorrelated in the highly negatively curved rim of the leading edge (Supplementary Fig. 15a, b). Here, Snx33 levels decreased while WAVE2 accumulated (Supplementary Fig. 15c, d), consistent with Snx33 being excluded from outward-curved regions and restricting WAVE-binding to such areas. Moreover, when cells collided, Snx33 localized to the cell contact area, whereupon WAVE2 disappeared and relocated to contact-free zones of the plasma membrane (Fig. 4o, p). Subsequently, the cell repolarized by forming a new leading edge (Fig. 4o, p). To assess whether the dysfunctional CIL response in Snx33-/- cells is due to impaired WAVE2 inhibition at the contact area, we followed WAVE2 localization before and after collision in Snx33-/- cells (Supplementary Videos 10). In contrast to wild-type cells, Snx33-/- cells indeed failed to remove WAVE2 from the contact site (Fig. 4q and Supplementary Fig. 15e). Altogether, we show a key role for the curvature-sensing protein Snx33 in regulating actin polymerization during CIL. Specifically, Snx33 displaces WAVE from cell–cell contact areas and thus leads to cell repolarization towards a contact-free zone upon collision with an obstacle.

## Discussion

Object evasion is key not only for the migration of immune and cancer cells in complex tissue environments, but also fundamental during embryogenesis and collective migration in vivo^49^. Combining genetic perturbations, microscopy, and microfluidics we identified a curvature-sensing mechanism underlying adaptive motility in immune-like cells. We show that the BAR domain protein Snx33 reads out changes in the membrane curvature landscape and feeds back to the actin polymerization machinery to regulate complex patterns at the leading edge of migrating cells. Specifically, Snx33 inhibits actin polymerization and tunes the localization of WAVE2, the main nucleation-promoting factor in neutrophils. Thereby, Snx33 limits the persistence of the leading edge and reduces migration directionality. This mechanism for promoting spontaneous reorientation during cell migration complements the one described for abundantly expressed I-BAR domain proteins, where outward curvature-sensing coupled to local activation of actin polymerization enhances exploratory cell behaviors^22^. While few other BAR domain proteins contain domains with inhibitory effects on actin polymerization, they are likely not fully redundant. We envision that their mechanism of action will be the result of several properties, including curvature sensitivity, strength of binding, binding partners, and what other domains are present. Thus, BAR domain proteins emerge as versatile tools that locally activate or inhibit actin polymerization and thus endow cells with functional control of their leading edge.

The ability to spontaneously reorient and explore the microenvironment with dynamic protrusions in and of itself contributes to more efficient navigation in crowded contexts^6,22,44,46,50,51^. A curvature-dependent downregulation of protrusive activity can furthermore aid object evasion more directly, by steering the cell away from the direction in which the presence of an obstacle on the outside is most likely. Indeed, we show that Snx33 is key for cells to circumnavigate both inert and cellular obstacles and thus migrate through complex three-dimensional environments. To date, little is known about the molecular machinery orchestrating CIL. srGAP2, a BAR domain protein that binds outward membrane curvature, was shown to induce protrusions during CIL^52–55^. Our study shows a key role for the inward curvature-sensing protein Snx33 in inhibiting actin polymerization during CIL. Specifically, we show that the multi-domain protein Snx33 localizes to cell–cell contacts and redistributes WAVE2 to the free surfaces, thereby reorienting the cell. This likely occurs in a curvature-dependent manner as Snx33 preferentially localizes to inward-curved membrane regions while WAVE2 is restricted to outward-curved areas at the leading edge^23^. At cell–cell contacts we identify a distinct shift in the curvature distribution using two datasets that differ in measured curvature values by an order of magnitude. Specifically, upon collision, the curvature distributions are narrower compared to that of the free leading edge of migrating single cells, consistent with membrane flattening upon collision. The expected curvature sensitivity of an Snx33 PX-BAR dimer corresponds to a higher curvature than that measured at cell–cell collisions. However, it is important to note that BAR domain proteins have auxiliary domains and can form oligomers as well as linear aggregates that bind membranes with considerably lower curvature^56–58^. Furthermore, our observations in cells are restricted to radii of curvature with magnitudes above the respective image resolution limits and limited by detectable curvature length scales. Thus, in the future, it will be important to explore how forming assemblies as well as the presence/absence of auxiliary domains may permit BAR domain proteins to span larger radii of curvatures to respond to larger-scale changes in membrane shape within the molecular complexity of cells^19,59^.

Here, we show that regulation of actin polymerization by Snx33 has important consequences for cell migration. This, in conjunction with the inherent curvature-sensing properties of BAR domain proteins and the observed shift in membrane curvature distribution upon cell–cell collision during CIL suggest a mechanism by which membrane curvature changes direct adaptive cellular behaviors. Together, our study supports the notion that cells use their membrane topography to encode information about the external environment they encounter.

Given the diversity of BAR domain proteins present in cells, we expect that this regulatory principle is likely to be used in many biological functions that have to react to shape changes, from the subcellular level (organelle homeostasis, membrane trafficking), via the single-cell level (immune surveillance, tumor dissemination), to the multicellular level (gastrulation, tissue folding).

## Methods

### Cell culture

HL-60 cells were grown in RPMI 1640 media with 10% heat-inactivated FBS (#10500-064, Gibco) and 1% Penicillin-Streptomycin (#15140-122, Gibco) in a humidified incubator at 37 °C with 5% CO_2_. Cells were differentiated by adding 1.5% DMSO (#D2438, Sigma Aldrich) and used after 5 days. Each independently differentiated batch was treated as a biological replicate. For starvation, cells were kept for 1 h in FBS-free RPMI 1640 media with 0.3% fatty acid-free BSA (#A7030-10G, Sigma Aldrich). For imaging or fixation, dHL-60 cells were plated on fibronectin-coated (0.01 mg/ml, #356008, Corning) glass-bottom dishes (#627860, Greiner bio-one) and allowed to adhere for 10 min in growth media. Next, cells were washed and stimulated with 10 nM fMLP (#F3506-5MG, Sigma Aldrich). For lowering adhesion, the coating was supplemented with 5% molar BSA (#A7030-10G, Sigma Aldrich). To generate stable cell lines with fluorescently tagged Snx33 and its truncations (PXBAR and ΔPXBAR), as well as Hem1 and CAAX lentiviral transduction was used as described previously^44^. Cells were sorted on a BD FACS Aria™ Fusion at the EMBL flow cytometry core facility.

### Generation of knockout cell line by CRISPR/Cas9

CRISPR/Cas9 generation in HL-60 cells was performed as described previously^45^. Briefly, cloning of the target guide sequence to target *Snx33* was performed^60,61^ (Forward: *CACCGctgggacgacGGATGCACAG*; Reverse: *aaacCTGTGCATCCgtcgtcccagC*). Cells expressing BFP-tagged Cas9 were single-cell sorted in 96-well plates on a BD FACS Aria^TM^ Fusion at the EMBL flow cytometry core facility. Single-cell clones were verified by genomic DNA amplification by Touchdown PCR^62^ and sequencing, followed by a Western blot of selected clonal lines.

### RNA sequencing

Total RNA samples obtained from 3 biological replicates were purified using a RNeasy Mini Kit (#74104, Qiagen) according to the manufacturer’s instructions with a DNase digestion step (#79254, Qiagen). To ensure high quality, samples were analyzed on an Agilent 2100 Bioanalyzer (Agilent Technologies). RNA sequencing was performed on an Illumina NextSeq 500 platform as NextSeqHigh-75 SE at the EMBL genomics core facility. For sequence alignment, the hg19 reference genome was used. Differential expression analysis was performed with a custom-made Galaxy pipeline using a DESeq2 package. The RNAseq data have been deposited to the ArrayExpress collections from BioStudies with the accession number E-MTAB-12436.

### Imaging

TIRFM images of live cells were acquired on a Nikon Ti Eclipse inverted microscope with a CFI Plan Apo Lambda 100x Oil (#MRD01905, Nikon) silicone objective and a sCMOS camera controlled by NIS-Elements (Nikon). Sample drift was reduced using an autofocus system (Perfect Focus, Nikon) for time-lapse imaging.

Confocal images of fixed cells were obtained with a UPLSAPO 60X S (NA 1.3; WD 0.3 mm) silicone objective on an Olympus FV3000 inverted microscope at the EMBL advanced light microscopy facility.

Epifluorescent and bright-field imaging of fixed cells was performed using a 40x objective (#MRD00405, Nikon), a SOLA SE II, and 100 W halogen lamps (Nikon) using appropriate filter sets.

Polarized TIRFM (pTIRFM) modality was implemented based on previous work^63–68^. For imaging, dHL-60 cells were stained before plating with carbocyanine dye DiI (#D3911, ThermoFisher Scientific).

Lattice light sheet imaging of live cells was performed on a Zeiss Lattice Light sheet 7 (Zeiss, Oberkochen, Germany) using appropriate filter sets and a 10 × 550 μm base beam.

### Image analysis

For confocal images (Fig. 1g and Supplementary Fig. 15b), only the *z*-planes that contained the top 80% intensity of mCherry-CAAX were considered based on line scans covering the entire resliced maximum intensity z projection. A channel of interest (ChoF1) was used for mask generation based on automatic Otsu segmentation. A custom-made ImageJ script allowed us to calculate the Pearson correlation coefficient (PCC) for every *z*-plane of ChoF1 with ChoF2 based on the mask of ChoF1 using the in-built Coloc2 ImageJ plugin. *Z*-slices were assigned to 10 bins and the mean with standard error of the mean for every bin was calculated.

For analysis of migrating cells imaged by TIRFM (Fig. 2b–f, Fig. 3b–d, and Fig. 4c–e) a segmentation of the cell mask (based on mCherry-CAAX signal) and of the WAVE2 mask (based on eGFP-Hem1 signal) were acquired using the machine-learning-based ilastik software (Versions 1.3.1b1 and 1.3.3post3)^27^. Further image analysis was achieved using an in-house built program implemented in Python. The angle at which cells are moving was calculated based on the center of mass for three consecutive frames. The leading edge was defined as the difference between two consecutive frames where at least one pixel of WAVE2 mask is present per cluster. The leading edge length was defined as the number of pixels in the outside perimeter of the leading edge. For the analysis of cell–cell contacts the same segmentation strategy was used to segment individual cells. Cell–cell contact was defined as the perimeters’ intersection of both cells.

For membrane topography analysis of SEM data (Fig. 3e, f, Supplementary Fig. 8j, Supplementary Fig. 11a–c), the leading edge area with ruffles was manually segmented on median filtered images. Next, ridges were detected within the segmented regions using the Meijering filter^69^. Ridges were later segmented using automatic Otsu thresholding and skeletonized using a custom Python script. Inversion of the number of pixels within the skeletonization per leading edge area corresponds to the effective ruffle wavelength.

For analysis of membrane curvature of SBEM and lattice light sheet images (Fig. 4i–n and Supplementary Fig. 13b–e, g, i), the plasma membrane was manually segmented at the leading edge (defined as the area in front of the nucleus) or segmented using Otsu threshold on the fluorescence channel with membrane signal followed by a manual curation, respectively. Next, the curvature was quantified by a circle fitting to a window with a user-defined length (2 times the specified half-width + 1 pixel) sliding along the segmented membrane, using a custom Python script. For determining the sign of the curvature, fiducials were manually added to the images to point to the cell interior (Supplementary Fig. 14a–d).

For analysis of protein position in relation to membrane based on lattice light sheet images (Fig. 1m and Supplementary Fig. 5) only cells with visible membrane ruffles in MIP were included in the analysis. The ruffles were segmented in the MIP by Mejiering filtering followed by Otsu thresholding. For all pixels in the user-defined ROI *z*-lines of the membrane and Snx33 channels were extracted and normalized to their peak intensity. To account for changes in cell thickness at different *x*–*y* positions the *z*-lines were normalized by localizing the peaks in the membrane channel corresponding to the top and bottom cell membranes (all *z*-lines with more than 2 peaks detected are excluded from further analysis). The normalized *z*-lines were then separated into two halves corresponding to the bottom and top cell membrane, each of the halves was then assessed for Snx33 colocalization by calculating the overlap between the Snx33 and membrane channels. All *z*-line halves showing low overlap are excluded from further analysis. Next, all *z*-lines corresponding to one cell were pooled and separated into the in-ruffle and off-ruffle groups as well as the top- and bottom-membrane subgroups. To allow unbiased comparison between the in-ruffle and off-ruffle groups the distribution of *z*-lines in each group was adjusted to contain the same frequency of cell thicknesses. In practice, as the off-ruffle group contains more *z*-lines than the in-ruffle group the off-ruffle group was randomly subsampled to reconstitute the distribution in the in-ruffle group. Finally, as the SNR of single *z*-lines does not allow for accurate assessment of Snx33 to membrane distance we employ averaging by bootstrap where random 100 *z*-lines from each group were averaged and renormalized to their peak intensity. The distance was then measured between the positions where the signal first reaches 20% in the membrane and Snx33 channels. This process was then repeated multiple times to generate a distribution of distances that captures the heterogeneity of raw data. For each cell, the mean value for each of the 4 subgroups was extracted and they were compared to validate the robustness of the approach.

### Cell migration assays in PDMS-based devices

PDMS-based microfluidic devices were prepared as previously described^46,70,71^. The devices used for the migration of dHL-60 cells had heights of 2.8 and 3.13 μm for channels with decision points and channels with constriction, respectively. The decision channels had constrictions of 2, 3, 4, and 5 μm in two arrangements. The channels with single constrictions were 2 μm. To visualize nuclei and cell body, Hoechst 33342 (#62249, Thermo Fisher Scientific) and TAMRA (Invitrogen) were added before the introduction of cells into the PDMS device. Cell migration towards chemoattractant (fMLP) was imaged on an inverted wide-field Nikon Eclipse microscope using a 20x/0.5 PH1 air objective, equipped with a Lumencor light source (390 nm, 475 nm, 542/575 nm), an incubation chamber and a heated stage with CO_2_. The acquired data were analyzed using ImageJ and manually curated. Only single cells that moved through the entire channel were considered for analysis. All parameters were quantified based on the nuclei signal.

### Tether extrusion using atomic force spectroscopy

Apparent membrane tension was measured by extruding plasma membrane tethers. For measurements, a Olympus BioLever (*k* = 60 pN/nm) from Bruker was mounted on a CellHesion 200 AFM (Bruker) with JPK SPM Software 6.1.183, which is integrated into an Eclipse Ti inverted light microscope (Nikon). Cantilevers were calibrated using the thermal noise method and coated with 2.5 mg/ml Concanavalin A (#C5275, Sigma Aldrich). Prior to the measurements, cantilevers were rinsed in dPBS. For tether measurement, the cantilever was positioned over the cell, preferably over the leading edge. Measurements parameters for static tether pulling experiments were as follows: approach velocity was set to 1 μm/s, contact force to 100–300 pN, contact time to 5–10 s, and retraction speed to 10 μm/s. After a 10 μm tether was pulled, the cantilever position was held constant until it broke, but no longer than 30 s. In every experimental repetition, the conditions’ order was randomized. For every cell at least 3 different tether measurements were taken.

The data analysis was performed using the JPK Data Processing Software 6.1.183. For assessing the magnitude of membrane tension based on tether force measurements, the following formula was used^44^:

1 $$T=\frac{{F}_{0}^{2}}{8B{\pi }^{2}}$$

where *F*_0_ is the tether force measured by the AFM and *B* is the bending rigidity of the plasma membrane, which we assume to be invariable between different experimental conditions (2.7 × 10^−19^ Nm based on previous measurements^72,73^.

### F-actin staining of non-adherent dHL-60 cells upon fMLP stimulation

Cells were starved and fixed by adding an equal volume of 2x fixation buffer to 10^6^ cells in growth media or stimulated with 10 nM fMLP and fixed 0-, 1-, and 30-min post-stimulation. Fixation buffer (1x) contains 3.7% paraformaldehyde (#28908, Thermo Scientific), 1x intracellular buffer (140 mM KCL, 1 mM MgCl_2_, 2 mM EGTA, 20 mM HEPES, pH 7.5), 320 mM sucrose (#S0389-500G, Sigma Aldrich) and 0.2% BSA (#A7030-10G, Sigma Aldrich). Next, cells were washed carefully with 1x intracellular buffer by plate centrifugation and stained with phalloidin coupled with TRITC (#P1951, Sigma Aldrich) for 1 h in intracellular buffer (1x) containing 0.2% of Triton X-100 (#T8787, Sigma Aldrich). Cells were then again washed with intracellular buffer (1x). Finally, they were re-suspended in 1 ml of 0.1% BSA, 2.5 mM EDTA in dPBS, and analyzed using a Cytek® Aurora (Cytek) at the EMBL Flow Cytometry Core Facility. Data were further analyzed and plotted using the FlowJo software (Version 10.9.0).

### Co-immunoprecipitation

For coimmunoprecipittion, GFP-tagged Snx33 and its truncation were added to the Snx33-/- cell line by viral transduction followed by cell sorting. Harvesting and lysis of dHL-60 cells was performed as recommended for cytoplasmic proteins following the ChromoTek GFP-Trap Magentic Particles M-270 with protease inhibitor supplementation (#gtd, Chromotek). GFP-nanotrap beads were used to precipitate GFP-tagged proteins from the lysate (full-length Snx33 and two truncations: PXBAR and ΔPXBAR) after overnight rotation. Elution was performed in 2xSDS-sample buffer and submitted for mass spectrometry.

### Protein mass spectrometry

#### Sample preparation

Reduction and alkylation were performed with dithiothreitol (56 °C, 30 min, 10 mM in 50 mM HEPES, pH 8.5) and 2-chloroacetamide (room temperature, in the dark, 30 min, 20 mM in 50 mM HEPES, pH 8.5). Samples were prepared according to the SP3 protocol (10.15252/msb.20145625, 10.1038/s41596-018-0082-x). In short, sequencing-grade trypsin (Promega) was added in an enzyme-to-protein ratio of 1:50 for overnight digestion at 37 °C. Peptide recovery was performed in 50 mM HEPES, pH 8.5 by collecting the supernatant on the magnet and combining it with a second elution.

Peptides were labeled with TMT16plex Isobaric Label Reagent (ThermoFisher) according to the manufacturer’s instructions. In short, 0.8 mg reagent was dissolved in 42 µl acetonitrile (100%) and 8 µl of stock was added and incubated for 1 h at room temperature. The reaction was quenched with 5% hydroxylamine for 15 min at RT. Samples were combined and cleaned up with an OASIS® HLB µElution Plate (Waters).

#### Mass spectrometry analysis

An UltiMate 3000 RSLC nano-LC system (Dionex) fitted with a trapping cartridge (µ-Precolumn C18 PepMap 100, 5 µm, 300 µm i.d. x 5 mm, 100 Å) and an analytical column (nanoEase™ M/Z HSS T3 column 75 µm x 250 mm C18, 1.8 µm, 100 Å, Waters) was coupled to an Orbitrap Fusion™ Lumos™ Tribrid™ Mass Spectrometer (Thermo) using the Nanospray Flex™ ion source in positive ion mode. Peptides were concentrated with a constant flow rate of 30 µl/min (0.05% trifluoroacetic acid in water) onto the trapping column for 4 min. Subsequently, peptides were eluted via the analytical column running using solvent A (0.1% formic acid in water, 3% DMSO) with a constant flow of 0.3 µl/min and with an increasing percentage of solvent B (0.1% formic acid in acetonitrile, 3% DMSO) from 2% to 8% in 4 min, from 8% to 28% for a further 104 min, in another 4 min. from 28% to 40%, and finally 40%–80% for 4 min followed by re-equilibration back to 2% B in 4 min.

MS instrument parameters were as follows: spray voltage of 2.4 kV, capillary temperature 275 °C, MS1 mass range 375–1500 *m*/*z*, profile mode, in the orbitrap with a resolution of 120000. The maximum fill time 50 ms, with an AGC target set to standard. Data-dependent acquisition (DDA) was performed with the resolution of the Orbitrap set to 30000, with a fill time of 94 ms and a limitation of 1 × 10^5^ ions. A normalized collision energy of 34 was applied. MS2 data was acquired in profile mode. Fixed first mass at 110 *m*/*z*.

#### Mass spectrometry data analysis—Isobarquant

IsobarQuant^74^ and Mascot (v2.2.07) were utilized to process the acquired data. Data was searched against the Homo sapiens proteome database (UP000005640) containing common contaminants and reversed sequences. The following modifications were included in the search parameters: Carbamidomethyl (C) and TMT10 (K) (fixed modification), Acetyl (Protein N-term), Oxidation (M) and TMT10 (N-term) (variable modifications). For the full scan (MS1) a mass error tolerance of 10 ppm and for MS/MS (MS2) spectra of 0.02 Da was set. Further parameters were set: Trypsin as protease with an allowance of a maximum of two missed cleavages: a minimum peptide length of seven amino acids; at least two unique peptides were required for protein identification. The false discovery rate on peptide and protein levels was set to 0.01.

Raw output files of IsobarQuant (protein.txt–files) were analyzed using R programming language (ISBN 3-900051-07-0). Only proteins with at least two unique peptides were included in the analysis and quantification. In total, 561 proteins passed the quality control filters. Raw signal-sums (signal_sum columns) were cleaned for batch effects using limma (PMID: 25605792) and later normalized using vsn (variance stabilization normalization-PMID: 12169536). To test proteins for differential enrichment limma package was employed. The replicate information was appended as a factor in the design matrix given as an argument to the ‘lmFit’ function of limma. A hit was defined as a protein annotated with a false discovery rate (fdr) smaller 5% and a fold-change of at least 100% and as a candidate with a fdr below 20% and a fold-change of at least 50%. The mass spectrometry proteomics data have been deposited to the ProteomeXchange Consortium via the PRIDE^75^ partner repository with the dataset identifier PXD033666.

### MD simulations

#### Coarse-grained molecular dynamics simulations

Molecular dynamics simulations were performed using GROMACS 2021.4^76^, using the coarse-grained Martini2.2 force field^77,78^ and applying an established scaling procedure (alpha = 0.7)^79^ to all protein beads.

As a basis for the structural model, an existing crystal structure (PDB ID: 4AKV) of the Snx33 membrane-binding domain (PX-BAR) was used. Since some loops were missing in this structure we predicted the same sequence using AlphaFold-multimer^80,81^ with default settings but increasing the number of recycles to 6. The resulting model was in excellent agreement (RMSD: 2.3 Å) with the crystal structure and was directly used for simulations. The structural model was coarse-grained using the ‘martinize.py’ script^78^ applying secondary structure restraints assigned by DSSP and using an elastic network^82^ across both subunits with *f*_c_ = 500 kJ/mol^−2^ and a cut-off *c* = 1.2 nm as previously reported for simulations of other extended BAR proteins^19^. For all disordered coil regions, all elastic bonds were removed.

The protein was then placed onto a buckled membrane which was generated from compression of a flat membrane according to the following procedure. A flat symmetric bilayer with a size of 70 x 35 x 20 nm^3^ was prepared using the ‘insane.py’ tool^83^. A plasma membrane-derived lipid composition determined by mass spectrometry was used as input (Table S1). The membrane was solvated and Na^+^-ions were added to make the system charge neutral. The membrane was then energy minimized using the steepest decent algorithm for 1000 steps.

Subsequently, the bilayer was simulated in an NPT ensemble (semi-isotropic pressure coupling) for 50 ns with a timestep of 20 fs using the Berendsen barostat^84^ and velocity rescaling thermostat^85^. Coupling times of 1 ps and 4 ps were used, respectively. The temperature was set to 310 K in all simulations. The Verlet neighbor search algorithm was used to update the neighbor list, with the length and update frequency being automatically determined. Lennard-Jones and Coulomb forces were cutoff at *r*_c_ = 1.1 nm with the potential shifted to 0 using the Verlet-shift potential modifier^86^.

Then, lateral pressure was set to 10 bar to generate buckled membrane structures (*P*_x,y_ = 10 bar, *P*_z_ = 1 bar). Two membranes with different degrees of curvature were extracted from the trajectory of compression with a box size of 58.9 × 29.5 × 25.9 nm^3^ (excess membrane area = 25.1 nm^2^) and 56.3 × 28.2 × 28.3 nm^3^ (excess membrane area = 31.4 nm^2^).

In both systems, the protein was placed above the buckled membrane and re-solvated and charge-neutralized. A brief equilibration was performed using the above settings and maintaining position restraints on the protein with a *f*_c_ = 1000 kJ mol^−1^ nm^−2^. The equilibration was run for 250 ns. Production simulations were then carried out using anisotropic pressure coupling with fixed *x*,*y* dimensions and *P*_z_ = 1 bar. Production simulations were carried out for 30 µs. The same simulation parameters were used as above except the pressure was controlled using instead the Parrinello-Rahman barostat with a coupling time of 20 ps^87^.

Images and visualizations were made with VMD (Version 1.9.4)^88^. Analyses were carried out with MDAnalysis 1.1.1^89,90^ and python 3.6. To analyze the curvature preference of the protein on the buckled membrane a previously established protocol by Bhaskara et al.^17^ was used (see: https://github.com/bio-phys/MemCurv). A 2D Fourier expansion of a height function *h*(x,y) was fit to the phosphate beads of the membrane every 1 ns by optimizing a least-squared fit. Subsequently, the mean curvature H was derived from the shape operator of the fit height function *h*(*x*,*y*). The mean curvature H was calculated for the *x*,*y* position of the center of mass of the protein as well as a randomly selected phosphate bead position to sample the background membrane at every considered frame.

### Scanning electron microscopy (SEM)

After 30 min of 10 nM fMLP stimulation, cells were fixed in 2.5% GA (#16220, EMS) in 0,1 M PHEM buffer by adding 37 °C double strength fixative (5% GA in 0,1 M PHEM) directly 1:1 to the cell medium. After 10 min incubation, the fixative was replaced by fresh single-strength fixative, and cells were further fixed at room temperature for 1 h. After fixation, cells were washed 2 times in 0,1 M PHEM and 2 times in 0,1 M cacodylate buffer. Next, they were postfixed for 2 h on ice in freshly prepared and filtered 1% OsO_4_ (#19190, EMS) and 0.8% potassium ferrocyanide (K_4_[Fe(CN)_6_]*3H_2_O, #4984, Merck) in 0.1 M cacodylate buffer. After postfixation, the cells were washed 4 times in H_2_O, and left at 4 °C until further processing.

Next, cells were treated with freshly prepared and filtered 1% tannic acid (TA, CAS#1401-55-4, EMS) in water using a Pelco BioWave microwave for seven 1-min cycles alternating between 150 W and 0 W power. Steady temperature was set to 23 °C and the vacuum to on for all steps. After TA treatment, cells were washed 2x in H_2_O on the bench and 2x in H_2_O in the microwave for 40 s per step at 250 W power. Cells were then treated with 1% UA (#77870, Serva) in H_2_O using the same microwave program as for TA. After washing once in H_2_O and twice in 25% EtOH, cells were dehydrated in a graded series of ethanol (25%–50%–75%–90%–100%–100%) using a microwave program with a step length of 40 s and 250 W power, with a steady temperature at 4 °C and without vacuum. Finally, the cells were infiltrated with a graded series of Hexamethyldisilizane (HMDS, CAS# 999-97-3, Sigma Aldrich) in ethanol (25%–50%–75%–100%–100%) using a microwave program with 6 steps of 1 min each, with a power of 150 W for step 1, 3, 4 and 6, and 0 W for steps 2 and 5. After the final 100% HMDS infiltration, all HMDS was removed, and coverslips were left to dry overnight. Silica gel with moisture indicator (Merck) was added in 4 empty wells (corners) in the 24-well plate to remove excess humidity.

After drying, coverslips were mounted in aluminum stubs (Agar Scientific G301F) using carbon tape, and sputter coated with a layer of gold for 180 s at 30 mA current using a Quorum sputter coater model Q150RS.

Imaging was performed on a Zeiss Crossbean 540 microscope, using 5 kV acceleration voltage and 700 pA current for the electron beam, with a working distance of 5 mm. A secondary electron detector (SESI) was used for signal detection, and all images were acquired with a pixel size of 28,9 nm/pixel.

### Serial block-face scanning electron microscopy (SBEM)

After 30 min of 10 nM fMLP stimulation in a MatTek dish, cells were fixed in 2.5% GA (#16220, EMS) in 0.1 M PHEM buffer by adding 37 °C double strength fixative (5% GA in 0.1 M PHEM) directly 1:1 to the cell medium. After 10 min incubation, the fixative was replaced by fresh single-strength fixative, and cells were incubated in fixative at 4 °C overnight. After fixation, cells were washed 2 times in 0.1 M PHEM and 2 times in 0.1 M cacodylate buffer. Next, they were postfixed for 1.5 h on ice in 1% OsO_4_ (#19190, EMS) and 0.8% potassium ferrocyanide (freshly prepared and filtered) (K_4_[Fe(CN)_6_]*3H_2_O, #4984, Merck) in 0.1 M cacodylate buffer. After postfixation, the cells were washed 4 times in H_2_O, and left in H_2_O at 4 °C until further processing (5 days).

Next, the cells were stained in three consecutive steps in the following order: 1% Thiocarbihydrazide (TCH, #21900, EMS) in water, 2% OsO_4_ in water, and 1% UA (#77870, Serva) in water. For all three staining steps the cells were processed in a Pelco BioWave microwave with a 7-step program of 2 min each, with power alternating between 100 W and 0 W (starting with 100), steady temperature was set to 23 °C and vacuum on for all steps. Between each staining step, the cells were washed 4 times in H_2_O, two times on the bench, and two times in the microwave (40 s, 250 W power, vacuum off).

After UA staining, the cells were washed once in H_2_O and twice in 25% EtOH, then further dehydrated in the microwave in a graded ethanol series (50%–70%–90%–100%–100%). Microwave settings for the dehydration steps were: 40 s time, 250 W power, vacuum off, steady temp 4 °C.

After dehydration the cells were infiltrated in a graded series of durcupan resin (Durcupan ACM from Sigma, #44611-#44614 (four components)) in ethanol (25%–50%–75%–100%–100%–100% durcupan), using the microwave for 3 min per step at 150 W power, 23 °C steady temp and vacuum cycle.

Finally, the cells were put on a small amount of fresh resin (covering the center of the MatTek dish) and left for ~1–2 h to evaporate residual solvent and air bubbles. Before polymerization, most of the resin was removed (just enough to cover the center was left). A drop of durcupan was added to an 18 × 18 mm coverslip (to avoid trapping air bubbles), and the coverslip was put on top of the center of the MatTek dish. The assembly was polymerized in the oven at 60 °C for 2 days.

After polymerization, the MatTek dish was removed my sawing close to the coverslip, and the glass on both sides of the round center peace was removed by dipping the assembly in liquid nitrogen and warm water.

A small piece was cut out from the sample using a razor blade and mounted on a SEM pin (Micro to Nano Gatan 3View pins, #10-006003-50) using two-component silver conductive epoxy (Ted Pella, #16043). The sample was also covered with silver epoxy on the sides, and finally, sputter coated with a layer of gold for 180 s at 30 mA current using a Quorum sputter coater model Q150RS. For curing the silver epoxy, the sample was cured in total for an additional day at 60 °C.

Image acquisition was performed on a Zeiss GeminiSEM 450 with 3View from Gatan (DigitalMicrograph Version 3.51.3720.0; SmartSEM version 6.06 with Service Pack 4), with the program SBEM Image (2021.08.dev) installed for controlling the acquisition^91^. For image acquisition an acceleration voltage of 1.5 kV was used, a beam current of 300 pA, pixel size 10 nm and a dwell time of 1.6 µs. An on-point BSE detector was used for detection, with the contrast set to 99.9% and brightness to 11.8 (with inverted LUT), BSD bias −5 V. Slice thickness was set to 40 nm. Between each cycle an overview image was acquired at 165.76 nm pixel size and 0.8 µs dwell time.

### Statistical analysis

Statistical analyses were performed using R (Version 3.2.1), while data visualization by both R and Adobe Illustrator®. The normality of data distribution was tested by Shapiro–Wilk test. A two-tailed *t*-test was used for normal distribution. Otherwise, a non-parametric Mann–Whitney-*U*-test was used, if not indicated differently. In all box plots, the lower and upper hinges correspond to the first and third quartiles (the 25th and 75th percentiles). The upper whisker extends from the hinge to the largest value, but no further than 1.5*IQR (distance between the first and third quartiles). The lower whisker extends from the hinge to the smallest value, but no lower than 1.5*IQR of the hinge. Data beyond the end of the whiskers are plotted as black dots. The black line and dot correspond to the median and mean, respectively. All measurements were taken from distinct samples.

### Reporting summary

Further information on research design is available in the Nature Portfolio Reporting Summary linked to this article.

### Supplementary information

Supplementary Information

Description of Additional Supplementary files

Supplementary Video 1

Supplementary Video 2

Supplementary Video 3

Supplementary Video 4

Supplementary Video 5

Supplementary Video 6

Supplementary Video 7

Supplementary Video 8

Supplementary Video 9

Supplementary Video 10

Reporting Summary

### Source data

Source Data

## Data Availability

RCSB PDB database was used in the study with accession number 4AKV. The RNAseq data have been deposited to the ArrayExpress collections from BioStudies with the accession number E-MTAB-12436. The mass spectrometry proteomics data have been deposited to the ProteomeXchange Consortium via the PRIDE partner repository with the dataset identifier PXD033666. The MD simulation data are available through figshare [https://figshare.com/articles/dataset/Supporting_data_for_Sensing_their_plasma_membrane_curvature_allows_migrating_cells_to_circumvent_obstacles_by_Ewa_Sitarska_Silvia_Dias_Almeida_Marianne_Sandvold_Beckwith_Julian_Stopp_Jakub_Czuchnowski_Marc_Siggel_Rita_Roessner_Aline_Tschanz/22109204]. The raw numbers for charts and graphs are available in the Source Data file whenever possible. All other data and unique reagents that support this study are available from the corresponding authors upon request. Source data are provided in this paper.

## Code availability

The source code for MD simulations and image analysis are available through figshare [https://figshare.com/articles/dataset/Supporting_data_for_Sensing_their_plasma_membrane_curvature_allows_migrating_cells_to_circumvent_obstacles_by_Ewa_Sitarska_Silvia_Dias_Almeida_Marianne_Sandvold_Beckwith_Julian_Stopp_Jakub_Czuchnowski_Marc_Siggel_Rita_Roessner_Aline_Tschanz/22109204] and on github^92^ [https://github.com/JakubCzuchnowski/Sensing-their-plasma-membrane-curvature-allows-migrating-cells-to-circumvent-obstacles.git].

## References

[1] Sarris M, Sixt M (2015). Science direct navigating in tissue mazes: chemoattractant interpretation in complex environments. Curr. Opin. Cell Biol..

[2] Stoitzner P, Stössel H, Romani N, Pfaller K (2002). A close-up view of migrating langerhans cells in the skin. J. Investig. Dermatol..

[3] Weigelin B, Bakker G-J, Friedl P (2012). Intravital third harmonic generation microscopy of collective melanoma cell invasion: principles of interface guidance and microvesicle dynamics. Intravital.

[4] Diz-Muñoz, A. et al. Steering cell migration by alternating blebs and actin-rich protrusions. *BMC Biol.* 1–13 10.1186/s12915-016-0294-x (2016). 10.1186/s12915-016-0294-x PMC5010735 27589901

[5] Fritz-Laylin LK (2017). Actin-based protrusions of migrating neutrophils are intrinsically lamellar and facilitate direction changes. Elife.

[6] Leithner A (2016). Diversified actin protrusions promote environmental exploration but are dispensable for locomotion of leukocytes. Nat. Cell Biol..

[7] Baptista, D., Teixeira, L., van Blitterswijk, C., Giselbrecht, S. & Truckenmüller, R. Overlooked? Underestimated? Effects of substrate curvature on cell behavior. *Trends Biotechnol.* 1–17 10.1016/j.tibtech.2019.01.006 (2019). 10.1016/j.tibtech.2019.01.006 30885388

[8] Kessels MM, Qualmann B (2021). Interplay between membrane curvature and the actin cytoskeleton. Curr. Opin. Cell Biol..

[9] Carman PJ, Dominguez R (2018). BAR domain proteins-a linkage between cellular membranes, signaling pathways, and the actin cytoskeleton. Biophys. Rev..

[10] McMahon HT, Gallop JL (2005). Membrane curvature and mechanisms of dynamic cell membrane remodelling. Nature.

[11] Simunovic M, Voth GA, Callan-Jones A, Bassereau P (2015). When physics takes over: BAR proteins and membrane curvature. Trends Cell Biol..

[12] de Kreuk B-J, Hordijk PL (2014). Control of Rho GTPase function by BAR-domains. Small GTPases.

[13] Galic M (2012). External push and internal pull forces recruit curvature-sensing N-BAR domain proteins to the plasma membrane. Nat. Cell Biol..

[14] Zhao, W. et al. Nanoscale manipulation of membrane curvature for probing endocytosis in live cells. 1–9 10.1038/nnano.2017.98 (2017). 10.1038/nnano.2017.98 PMC5544585 28581510

[15] Lou H-Y (2019). Membrane curvature underlies actin reorganization in response to nanoscale surface topography. Proc. Natl Acad. Sci. USA.

[16] Hoogendijk AJ (2019). Dynamic transcriptome-proteome correlation networks reveal human myeloid differentiation and neutrophil-specific programming. Cell Rep..

[17] Bhaskara, R. M. et al. Curvature induction and membrane remodeling by FAM134B reticulon homology domain assist selective ER-phagy. *Nat. Commun.* 1–13 10.1038/s41467-019-10345-3 (2019). 10.1038/s41467-019-10345-3 PMC6542808 31147549

[18] Jensen LE (2022). Membrane curvature sensing and stabilization by the autophagic LC3 lipidation machinery. Sci. Adv..

[19] Mahmood MI, Noguchi H, Okazaki K-I (2019). Curvature induction and sensing of the F-BAR protein Pacsin1 on lipid membranes via molecular dynamics simulations. Sci. Rep..

[20] Bigay J, Antonny B (2012). Curvature, lipid packing, and electrostatics of membrane organelles: defining cellular territories in determining specificity. Dev. Cell.

[21] Mattila PK (2007). Missing-in-metastasis and IRSp53 deform PI(4,5)P 2-rich membranes by an inverse BAR domain–like mechanism. J. Cell Biol..

[22] Begemann, I. et al. Mechanochemical self-organization determines search pattern in migratory cells. *Nat. Phys.* 1–13 10.1038/s41567-019-0505-9 (2020).

[23] Pipathsouk A (2021). The WAVE complex associates with sites of saddle membrane curvature. J. Cell Biol..

[24] Weiner OD, Marganski WA, Wu LF, Altschuler SJ, Kirschner MW (2007). An actin-based wave generator organizes cell motility. PLoS Biol..

[25] Graziano BR (2017). A module for Rac temporal signal integration revealed with optogenetics. J. Cell Biol..

[26] Lacayo CI (2007). Emergence of large-scale cell morphology and movement from local actin filament growth dynamics. PLoS Biol..

[27] Berg S (2019). ilastik: interactive machine learning for (bio)image analysis. Nat. Methods.

[28] Gauthier NC, Fardin MA, Roca-Cusachs P, Sheetz MP (2011). Temporary increase in plasma membrane tension coordinates the activation of exocytosis and contraction during cell spreading. Proc. Natl Acad. Sci. USA.

[29] Houk AR (2012). Membrane tension maintains cell polarity by confining signals to the leading edge during neutrophil migration. Cell.

[30] Rocca DL, Martin S, Jenkins EL, Hanley JG (2008). Inhibition of Arp2/3-mediated actin polymerization by PICK1 regulates neuronal morphology and AMPA receptor endocytosis. Nat. Cell Biol..

[31] Cao H (2013). FCHSD1 and FCHSD2 are expressed in hair cell stereocilia and cuticular plate and regulate actin polymerization in vitro. PLoS ONE.

[32] Kostan J (2014). Direct interaction of actin filaments with F-BAR protein pacsin2. EMBO Rep..

[33] Dräger NM (2017). Bin1 directly remodels actin dynamics through its BARdomain. EMBO Rep..

[34] Chen P-W (2020). The BAR domain of the Arf GTPase-activating protein ASAP1 directly binds actin filaments. J. Biol. Chem..

[35] Graziano BR (2019). Cell confinement reveals a branched-actin independent circuit for neutrophil polarity. PLoS Biol..

[36] Akin O, Mullins RD (2008). Capping protein increases the rate of actin-based motility by promoting filament nucleation by the Arp2/3 complex. Cell.

[37] Cooper JA, Sept D (2008). New insights into mechanism and regulation of actin capping protein. Int. Rev. Cell Mol. Biol..

[38] Edwards, M. et al. Capping protein regulators fine-tune actin assembly dynamics. 1–13 10.1038/nrm3869 (2014). 10.1038/nrm3869 PMC4271544 25207437

[39] Funk, J. et al. A barbed end interference mechanism reveals how capping protein promotes nucleation in branched actin networks. *Nat. Commun.* 1–17 10.1038/s41467-021-25682-5 (2021). 10.1038/s41467-021-25682-5 PMC8429771 34504078

[40] Rao Y (2010). Molecular basis for SH3 domain regulation of F-BAR-mediated membrane deformation. Proc. Natl Acad. Sci. USA.

[41] Kast, D. J. et al. Mechanism of IRSp53 inhibition and combinatorial activation by Cdc42 and downstream effectors. 1–11 10.1038/nsmb.2781 (2014). 10.1038/nsmb.2781 PMC4091835 24584464

[42] Stanishneva-Konovalova TB (2016). Coordinated autoinhibition of F-BAR domain membrane binding and WASp activation by Nervous Wreck. Proc. Natl Acad. Sci. USA.

[43] Inagaki N, Katsuno H (2017). Actin waves: origin of cell polarization and migration?. Trends Cell Biol..

[44] Diz-Muñoz A (2016). Membrane tension acts through PLD2 and mTORC2 to limit actin network assembly during neutrophil migration. PLoS Biol..

[45] Graziano BR (2019). Cell confinement reveals a branched-actin independent circuit for neutrophil polarity. PLoS Biol..

[46] Renkawitz J (2019). Nuclear positioning facilitates amoeboid migration along the path of least resistance. Nature.

[47] Yamada, K. M. & Sixt, M. Mechanisms of 3D cell migration. *Nat. Rev. Mol. Cell Biol*. 1–15 10.1038/s41580-019-0172-9 (2019). 10.1038/s41580-019-0172-9 31582855

[48] Roycroft A, Mayor R (2015). Molecular basis of contact inhibition of locomotion. Cell. Mol. Life Sci..

[49] Stramer B, Mayor R (2016). Mechanisms and in vivo functions of contact inhibition of locomotion. Nat. Rev. Mol. Cell Biol..

[50] Driscoll MK (2012). Cell shape dynamics: from waves to migration. PLoS Comput. Biol..

[51] Stankevicins L (2020). Deterministic actin waves as generators of cell polarization cues. Proc. Natl Acad. Sci. USA.

[52] Coutinho-Budd J, Ghukasyan V, Zylka MJ, Polleux F (2012). The F-BAR domains from srGAP1, srGAP2 and srGAP3 regulate membrane deformation differently. J. Cell Sci..

[53] Guerrier S (2009). The F-BAR domain of srGAP2 induces membrane protrusions required for neuronal migration and morphogenesis. Cell.

[54] Fritz RD (2015). SrGAP2-dependent integration of membrane geometry and slit-robo-repulsive cues regulates fibroblast contact inhibition of locomotion. Dev. Cell.

[55] Ren C (2019). Leukocyte cytoskeleton polarization is initiated by plasma membrane curvature from cell attachment. Dev. Cell.

[56] Simunovic M, Srivastava A, Voth GA (2013). Linear aggregation of proteins on the membrane as a prelude to membrane remodeling. Proc. Natl Acad. Sci. USA.

[57] Simunovic M, Šarić A, Henderson JM, Lee KYC, Voth GA (2017). Long-Range Organization of Membrane-Curving Proteins. ACS Cent. Sci..

[58] Jarin Z (2019). Unusual organization of I-BAR proteins on tubular and vesicular membranes. Biophys. J..

[59] Nepal B, Sepehri A, Lazaridis T (2021). Mechanism of negative membrane curvature generation by I-BAR domains. Structure.

[60] Shalem O (2014). Genome-scale CRISPR-Cas9 knockout screening in human cells. Science.

[61] Sanjana, N. E., Shalem, O. & Zhang, F. Improved vectors and genome-wide libraries for CRISPR screening. *Nat. Meth.* 1–2 10.1038/nmeth.3047 (2014). 10.1038/nmeth.3047 PMC4486245 25075903

[62] Koch B (2018). Generation and validation of homozygous fluorescent knock-in cells using CRISPR–Cas9 genome editing. Nat. Publ. Group.

[63] Axelrod D (2013). Evanescent excitation and emission in fluorescence microscopy. Biophys. J..

[64] Anantharam A, Onoa B, Edwards RH, Holz RW, Axelrod D (2010). Localized topological changes of the plasma membrane upon exocytosis visualized by polarized TIRFM. J. Cell Biol..

[65] Oreopoulos J, Epand RF, Epand RM, Yip CM (2010). Peptide-induced domain formation in supported lipid bilayers: direct evidence by combined atomic force and polarized total internal reflection fluorescence microscopy. Biophys. J..

[66] Oreopoulos J, Yip CM (2008). Combined scanning probe and total internal reflection fluorescence microscopy. Methods.

[67] Sund SE, Swanson JA, Axelrod D (1999). Cell membrane orientation visualized by polarized total internal reflection fluorescence. Biophys. J..

[68] Axelrod, D. Chapter 7: Total internal reflection fluorescence microscopy. *Methods Cell Biol.* **89**, 169–221 (2008). 10.1016/S0091-679X(08)00607-9 19118676

[69] Meijering E (2004). Design and validation of a tool for neurite tracing and analysis in fluorescence microscopy images. Cytom. A.

[70] Renkawitz, J., Reversat, A., Leithner, A., Merrin, J. & Sixt, M. *Micro-engineered ‘pillar forests’ to study cell migration in complex but controlled 3D environments*. *Methods Cell Biol*. **147**, 79–91 (2018). 10.1016/bs.mcb.2018.07.004 30165964

[71] Kopf A (2020). Microtubules control cellular shape and coherence in amoeboid migrating cells. J. Cell Biol..

[72] Sens P, Plastino J (2015). Membrane tension and cytoskeleton organization in cell motility. J. Phys. Condens. Matter.

[73] Hochmuth FM, Shao JY, Dai J, Sheetz MP (1996). Deformation and flow of membrane into tethers extracted from neuronal growth cones. Biophys. J..

[74] Franken H (2015). Thermal proteome profiling for unbiased identification of direct and indirect drug targets using multiplexed quantitative mass spectrometry. Nat. Publ. Group.

[75] Perez-Riverol Y (2022). The PRIDE database resources in 2022: a hub for mass spectrometry-based proteomics evidences. Nucl. Acids Res..

[76] Abraham MJ (2015). GROMACS: high performance molecular simulations through multi-level parallelism from laptops to supercomputers. SoftwareX.

[77] Marrink SJ, Risselada HJ, Yefimov S, Tieleman DP, de Vries AH (2007). The MARTINI force field: coarse grained model for biomolecular simulations. J. Phys. Chem. B.

[78] Monticelli L (2008). The MARTINI coarse-grained force field: extension to proteins. J. Chem. Theory Comput.

[79] Benayad Z, Bülow, von S, Stelzl LS, Hummer G (2021). Simulation of FUS protein condensates with an adapted coarse-grained model. J. Chem. Theory Comput..

[80] Jumper, J. et al. Highly accurate protein structure prediction with AlphaFold. *Nature* 1–12 10.1038/s41586-021-03819-2 (2021). 10.1038/s41586-021-03819-2 PMC8371605 34265844

[81] Evans, R. et al. Protein complex prediction with AlphaFold-Multimer. *bioRxiv*10.1101/2021.10.04.463034 (2022).

[82] Periole X, Cavalli M, Marrink S-J, Ceruso MA (2009). Combining an elastic network with a coarse-grained molecular force field: structure, dynamics, and intermolecular recognition. J. Chem. Theory Comput.

[83] Wassenaar TA, Ingólfsson HI, Böckmann RA, Tieleman DP, Marrink SJ (2015). Computational lipidomics with insane: a versatile tool for generating custom membranes for molecular simulations. J. Chem. Theory Comput..

[84] Berendsen HJC, Postma JPM, van Gunsteren WF, DiNola A, Haak JR (1998). Molecular dynamics with coupling to an external bath. J. Chem. Phys..

[85] Bussi G, Donadio D, Parrinello M (2007). Canonical sampling through velocity rescaling. J. Chem. Phys..

[86] de Jong DH, Baoukina S, Ingólfsson HI, Marrink SJ (2016). Martini straight: Boosting performance using a shorter cutoff and GPUs. Comput. Phys. Commun..

[87] Parrinello M, Rahman A (1998). Polymorphic transitions in single crystals: a new molecular dynamics method. J. Appl. Phys..

[88] Humphrey W, Dalke A, Schulten K (1996). VMD: visual molecular dynamics. J. Mol. Graph.

[89] Gowers, R. et al. in *Proceedings of the 15th Python in Science Conference* (https://conference.scipy.org/proceedings/scipy2016/oliver_beckstein.html), pp. 98–105 (2016).

[90] Michaud-Agrawal N, Denning EJ, Woolf TB, Beckstein O (2011). MDAnalysis: a toolkit for the analysis of molecular dynamics simulations. J. Comput. Chem..

[91] Titze B, Genoud C, Friedrich RW (2018). SBEMimage: versatile acquisition control software for serial block-face electron microscopy. Front. Neural Circuits.

[92] Czuchnowski, J. Supporting data for “Sensing their plasma membrane curvature allows migrating cells to circumvent obstacles”. *GitHub*, 10.5281/zenodo.8169105 (2023). 10.1038/s41467-023-41173-1 PMC10499897 37704612

