# Supplementary material for: Sensing their plasma membrane curvature allows migrating cells to circumvent obstacles
Source: Nat Commun. 2023 Sep 13;14:5644. doi: 10.1038/s41467-023-41173-1 (PMC10499897; doi:10.1038/s41467-023-41173-1)
Supplement: Supplementary file 1 — Supplementary Information [file 41467_2023_41173_MOESM1_ESM.pdf]

Supplementary Information

Sensing their plasma membrane curvature allows migrating cells to circumvent obstacles

Ewa Sitarska[1,§], Silvia Dias Almeida[1,‡], Marianne Sandvold Beckwith[1], Julian Stopp[2], Jakub Czuchnowski[1], Marc Siggel[3,4], Rita Roessner[3,4], Aline Tschanz[1,§], Christer Ejsing[1,5], Yannick Schwab[1], Jan Kosinski[3,4,6], Michael Sixt[2], Anna Kreshuk[1], Anna Erzberger[1] and Alba Diz-Muñoz[1,*]

[1] Cell Biology and Biophysics Unit, European Molecular Biology Laboratory, 69117 Heidelberg, Germany.
[2] Institute of Science and Technology Austria, 3400 Klosterneuburg, Austria.
[3] EMBL Hamburg, European Molecular Biology Laboratory, 22607, Hamburg, Germany.
[4] Centre for Structural Systems Biology, 22607, Hamburg, Germany.
[5] Department of Biochemistry and Molecular Biology, Villum Center for Bioanalytical Sciences, University of Southern Denmark, Campusvej 55, 5230 Odense, Denmark.
[6] Structural and Computational Biology Unit, European Molecular Biology Laboratory, 69117 Heidelberg, Germany.
[6] Department of Biochemistry and Molecular Biology, Villum Center for Bioanalytical Sciences, University of Southern Denmark, Campusvej 55, 5230 Odense, Denmark.
[§] Collaboration for joint PhD degree between EMBL and Heidelberg University, Faculty of Biosciences.
[‡] Current address: Division of Medical Image Computing, German Cancer Research Center (DKFZ), 69120 Heidelberg, Germany.
[*] Correspondence: diz@embl.de

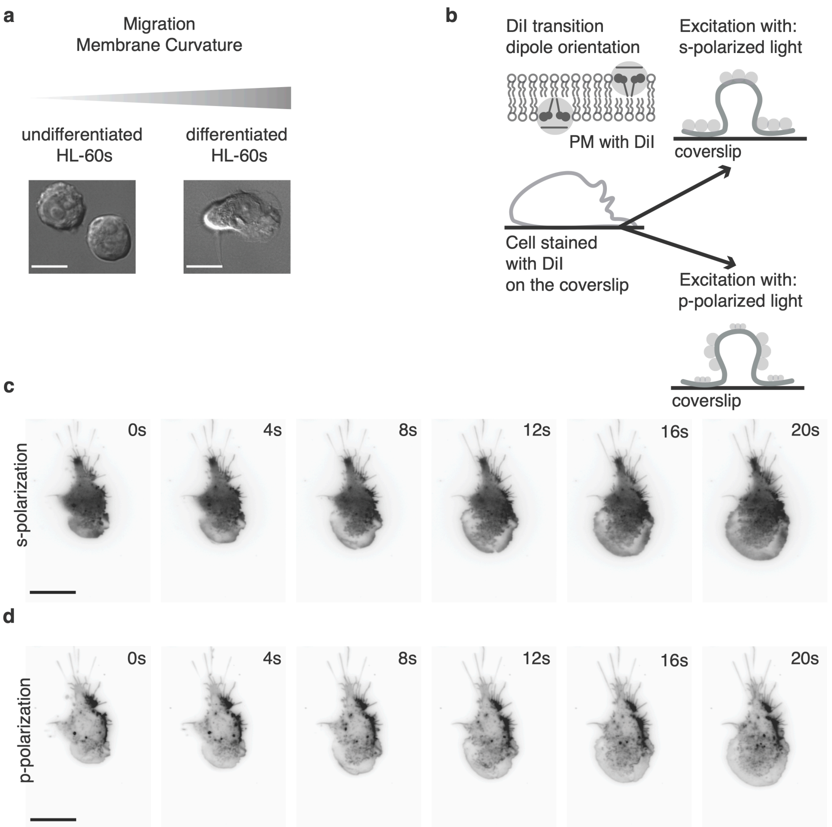

Supplementary Figure 1: Membrane topography imaged by pTIRFM. a) Schematic illustrating changes in HL-60 cells during differentiation. **b)** Schematic illustrating the principles of pTIRFM imaging using carbocyanine dyes (DiI). Time lapse pTIRFM imaging of a dHL-60 cell using **c)** s-polarization and **d)** p-polarization. Scale bars = 10 µm.

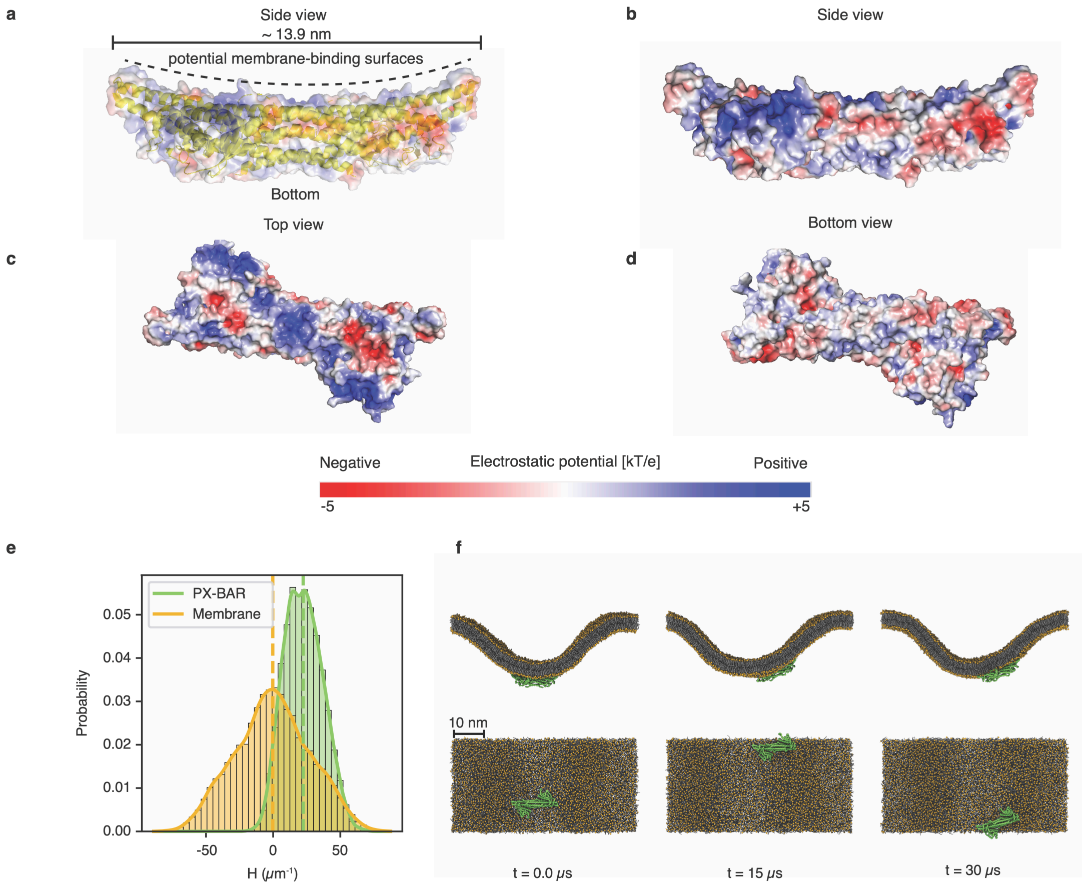

Supplementary Fig. 2: **Structure of the Snx33 membrane binding unit (PX-BAR) and MD simulations strongly suggest binding to positive (inward) membrane curvature. a)** Structure of PX-BAR domains of Snx33 (4AKV) together with electrostatic surface representation (side view). The dotted line indicates the potential membrane-binding surfaces, while the solid line shows overall dimensions of the dimer. **b-d)** Electrostatic surface representation of 4AKV side view **(b)**, top view **(c)** and bottom view **(d)**. Visualizations were created in PyMOL (The PyMOL Molecular Graphics System, Version 2.4.2 Schrödinger, LLC). The charges are shown on a scale from red (negative: -5) to blue (positive: +5)). **e)** Probability histogram of the mean curvature (H) sampled at the center of mass of PX-BAR (green) and at a random lipid phosphate position (yellow). Kernel density estimates to smooth the distributions are shown. The mean values are indicated as dashed vertical lines. **f)** Top and side views of snapshots from snapshots of the coarse-grained simulation of a buckled membrane with the PX-BAR domain with higher curvature than in **Fig. 1h-j** (excess area = 31.4 nm^2). See Methods for details. Protein backbone beads (green), phosphate beads (yellow spheres), and lipid tails (grey sticks) are shown. Water and ions are omitted for clarity. A top and side view are shown. Time points are indicated for the respective frames.

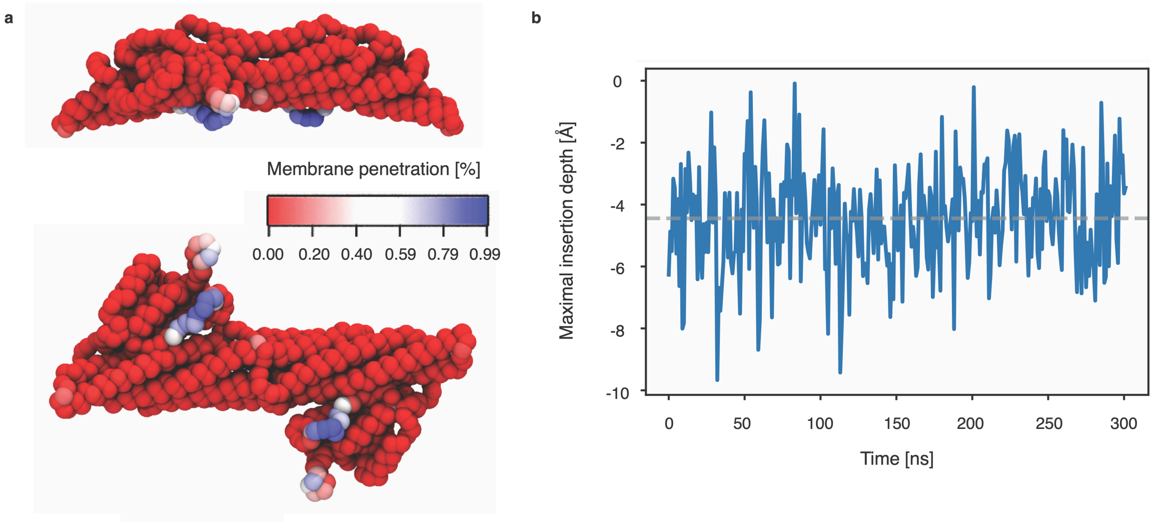

Supplementary Fig. 3: PX-BAR of Snx33 interacts with the plasma membrane by electrostatics with little insertion in the membrane.

a) Close-up views show how often each residue dips below the Fourier fit of the upper membrane leaflet proximal to Snx33 over the full trajectory. **b)** Maximal insertion depth of the protein considering all residues over time with respect to the Fourier fit. The insertion in the membrane is very shallow reaching the average of 4.3 Å (grey dashed line) and does not protrude significantly below the polar head group region.

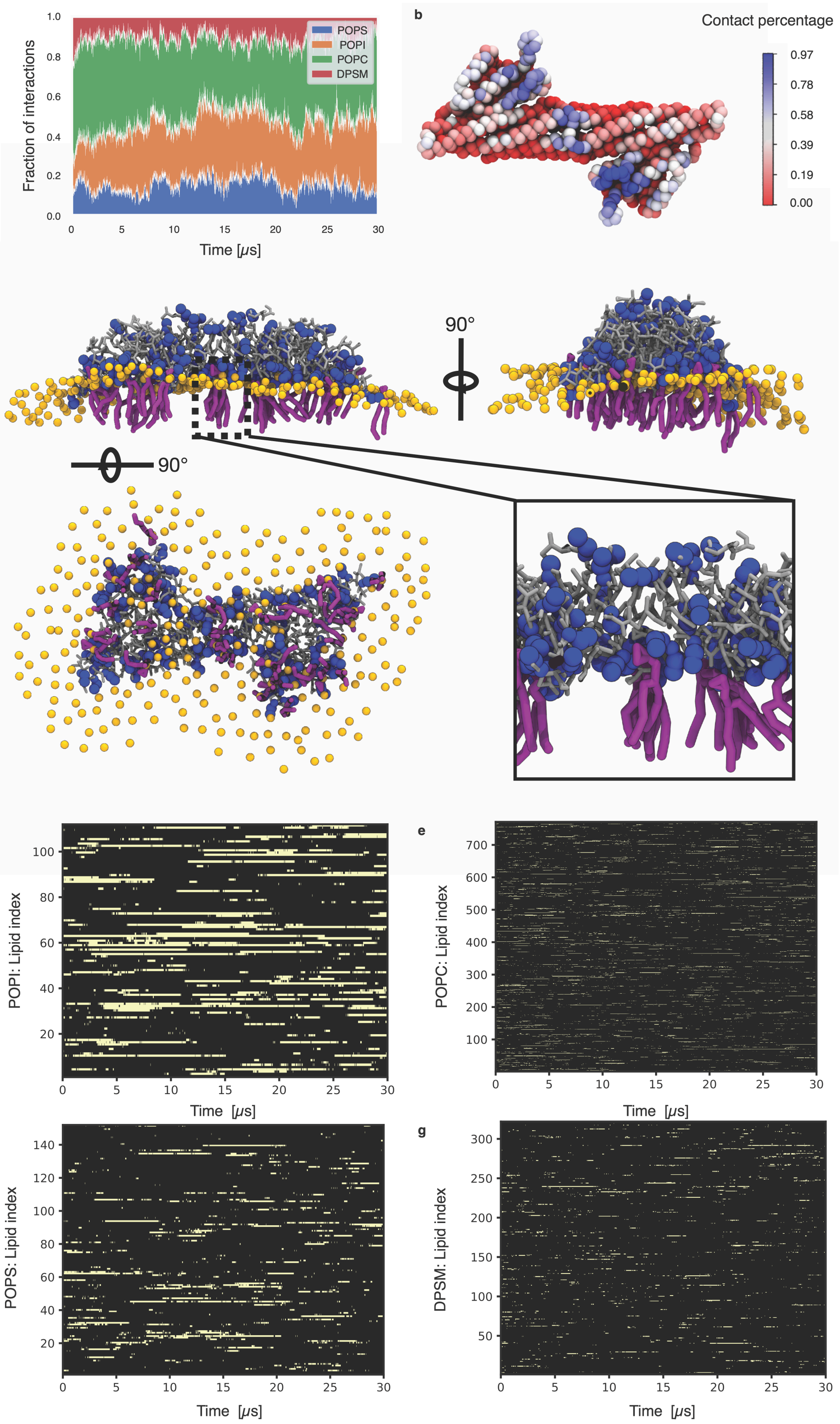

Supplementary Fig. 4: Specific interactions of Snx33 PX-BAR and lipids in the curved membrane. a) Stacked percentages graph shows lipid interactions with Snx33 PX-BAR depending on the lipid type (POPI, POPC, POPS, DPSM). Lipids are considered in contact if they are within 6 Å of the protein. **b)** Shown in the percentage of simulation time per residue in which Snx33 is in contact with any POPI lipids. The percentage per residue is mapped onto the backbone beads. **c)** Interactions of Snx33 PX-BAR on the membrane with specific POPI lipid. Bottom, side and a close-up views. Only the proximal leaflet is visualized. Snx33 does not submerge into the membrane. Lipids shown in purple, lysine and arginine residues are shown in blue. Other residues are shown in grey, PO4 beads in yellow. Lipid-protein residence times for **d)** POPI, **e)** POPC, **f)** POPS and **g)** DPSM. Each lipid is defined as interacting if within 8 Å of the protein. Each lipid in the upper leaflet is considered, while cholesterol is not considered.

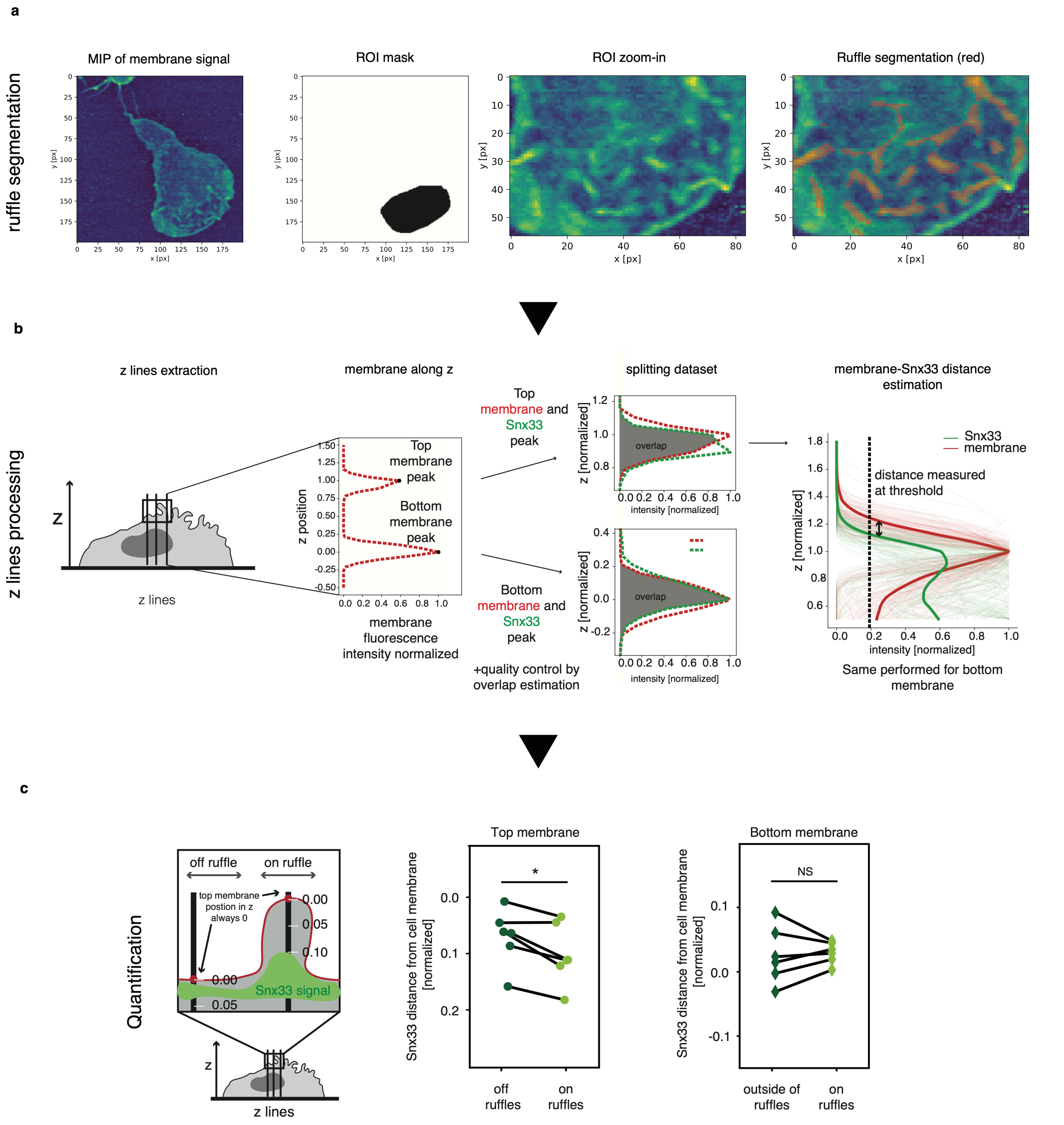

Supplementary Fig. 5: Workflow for analysis of two-color 3D lattice light sheet movies. a) Ruffle segmentation workflow. **b)** Z-lines were extracted for all pixels included in the leading edge segmentation from **a)** and processed by identifying both membranes followed by normalization and membrane-Snx33 distance estimation. **c)** All z-lines corresponding to one cells were pooled together for the off-ruffle and on-ruffle conditions and quantified ($p_{Top\ Membrane}$ = 0.01635, t = -3.5524, df = 5, paired T-test, two-sided; $p_{Bottom\ Membrane}$ = 0.7656, t = -0.31478, df = 5, paired T-test, two-sided). n= 6 cells. p<0.05 (*). MIP = Maximum Intensity Projection; ROI = Region of Interest.

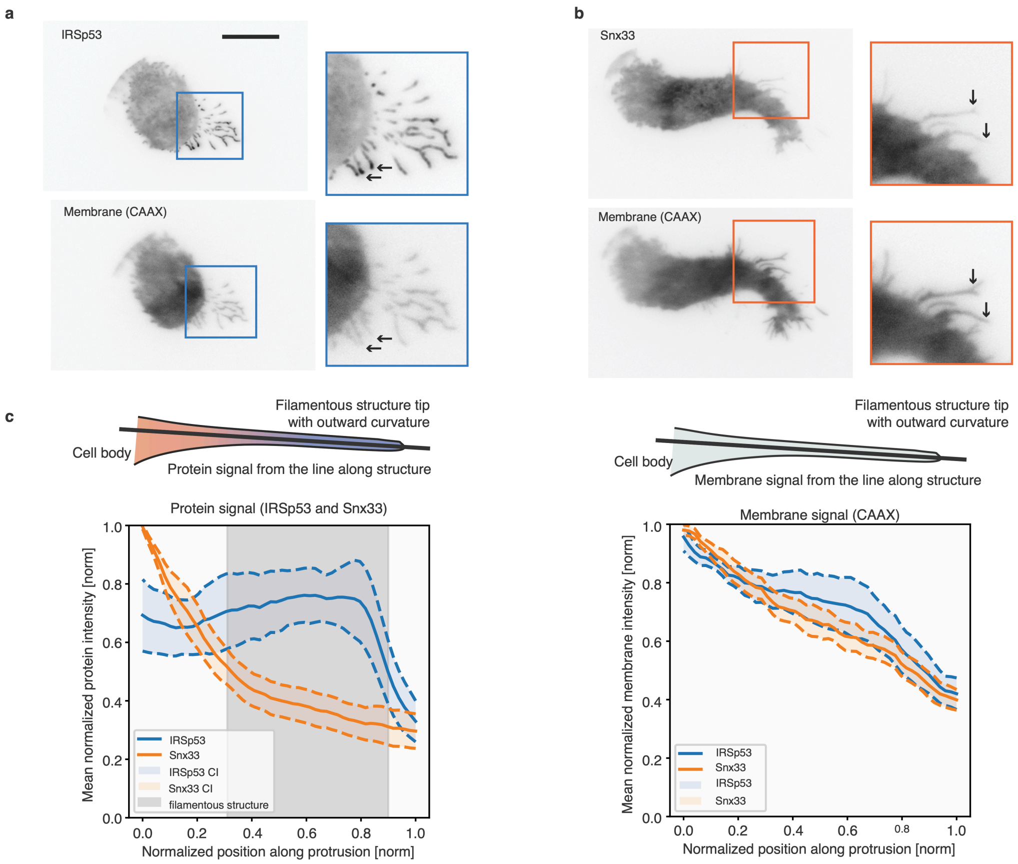

Supplementary Fig. 6: Distinct localization of positive (inward) binding Snx33 and negative (outward) binding IRSp53. a) Exemplary image of GFP-tagged IRSp53 (canonical outward sensing BAR domain protein) and membrane (CAAX) with zoom-in on filamentous structure tips (arrows) using TIRFM. **b)** Exemplary image of GFP-tagged Snx33 and membrane (CAAX) with zoom-in on filamentous structure tips (arrows) using TIRFM. **c)** Quantification of protein (IRSp53 and Snx33) and corresponding membrane signal along the filamentous structure. CI – confidence interval.

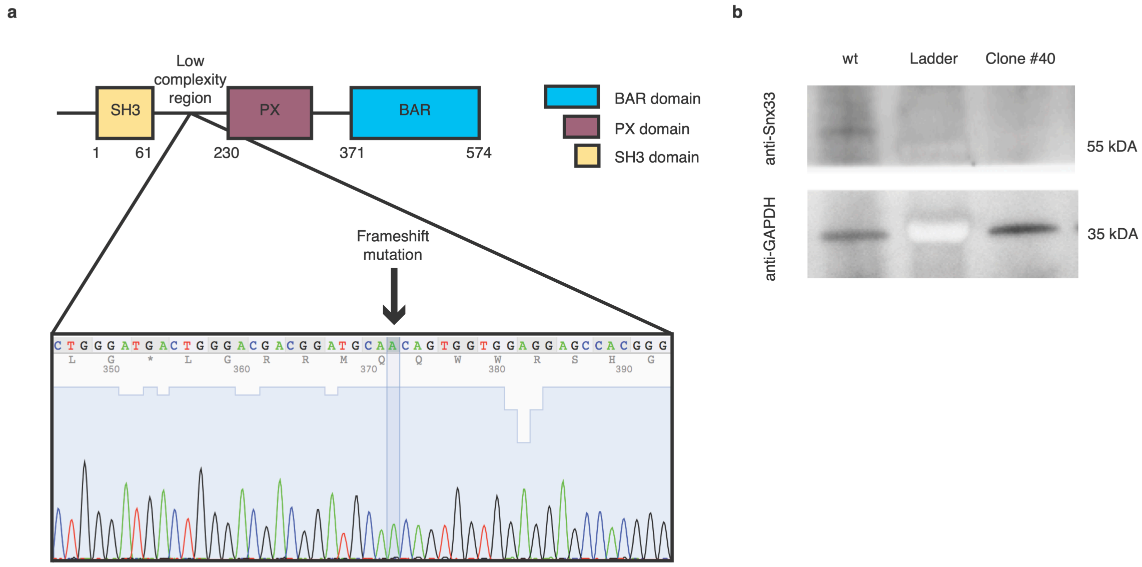

Supplementary Fig. 7: Snx33 knockout cell line validation. a) Snx33 clone sequencing confirming a frameshift mutation. **b)** Snx33 and GAPDH Western blots of wt cells and Snx33 -/- clone. n=3.

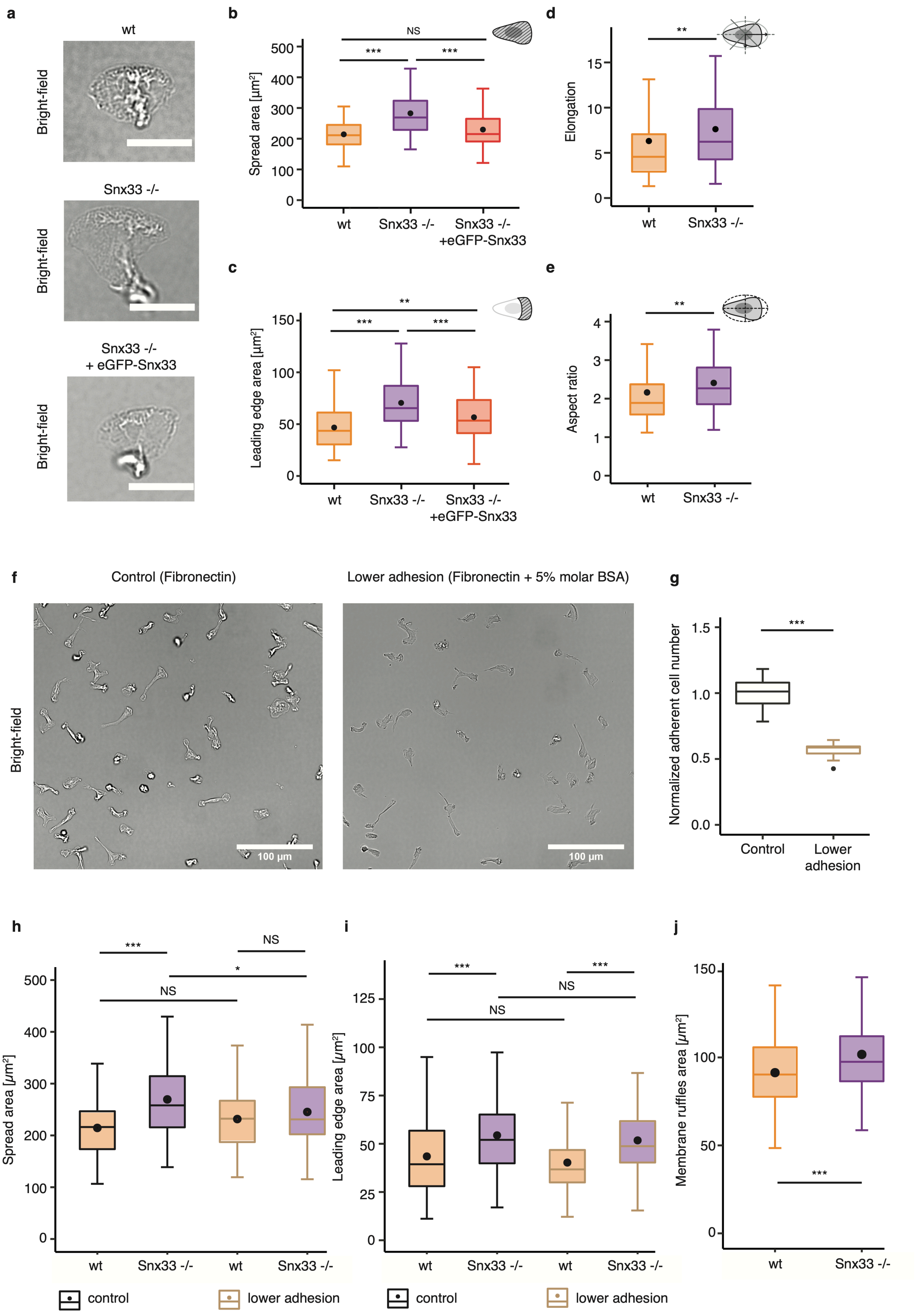
a
wt
Bright-field
Snx33 -/-
Bright-field
Snx33 -/-
+ eGFP-Snx33
Bright-field
b
Spread area [µm²]
NS
***
***
wt
Snx33 -/-
Snx33 -/-
+eGFP-Snx33
c
Leading edge area [µm²]
**
***
***
wt
Snx33 -/-
Snx33 -/-
+eGFP-Snx33
d
Elongation
**
wt
Snx33 -/-
e
Aspect ratio
**
wt
Snx33 -/-
f
Control (Fibronectin)
Lower adhesion (Fibronectin + 5% molar BSA)
Bright-field
100 µm
100 µm
g
Normalized adherent cell number
***
Control
Lower
adhesion
h
Spread area [µm²]
***
NS
*
NS
wt
Snx33 -/-
wt
Snx33 -/-
control
lower adhesion
i
Leading edge area [µm²]
***
***
NS
NS
wt
Snx33 -/-
wt
Snx33 -/-
control
lower adhesion
j
Membrane ruffles area [µm²]
***
wt
Snx33 -/-

Supplementary Fig. 8: Cell and leading edge morphology in Snx33 knockout and wt cells and their independence on adhesion strength. a) Example images of bright-field of wild-type, Snx33 -/- and Snx33 -/- with eGFP-tagged Snx33 cells. Quantification of **b)** cell spread ($p_{\text{wt vs Snx33 -/-}}$ = 2.474e-08; $p_{\text{wt vs Snx33 -/- + GFP-Snx33-/-}}$ = 0.1623; $p_{\text{Snx33 -/- vs Snx33 -/- + GFP-Snx33-/-}}$ = 1.233e-5, all Mann-Whitney-U-Test, two-sided) and **c)** leading edge area. Data from 3 independent experiments ($p_{\text{wt vs Snx33 -/-}}$ = 9.358e-9, Mann-Whitney-U-Test, two-sided; $p_{\text{wt vs Snx33 -/- + GFP-Snx33-/-}}$ = 0.009236, Mann-Whitney-U-Test, two-sided; $p_{\text{Snx33 -/- vs Snx33 -/- + GFP-Snx33-/-}}$ = 0.0005547, t = 3.5363, df = 136.51, two-sided). N_{wt} = 67, $n_{\text{Snx33-/-}}$ =73, $n_{\text{Snx33 -/- +GFP-Snx33}}$ = 67. **d)** Quantification of cell elongation (p = 0.004832, Mann-Whitney-U-Test, two-sided) and **e)** aspect ratio from TIRFM images. N_{wt} = 82, $n_{\text{Snx33 -/-}}$ = 78 (p = 0.004989, Mann-Whitney-U-Test, two-sided). **f)** Example bright-field images of wt dHL-60 cells on control (fibronectin only) and lower adhesion substrate (fibronectin with 5% molar BSA). **g)** Adhesion-dependent cell number quantification. Data from two independent experiments (p = 1.691e-7, t = 9.7419, df = 13.568, two-sided). N_{control} = 10, $n_{\text{lower adhesion}}$ = 10. Quantification of **h)** cell spread area ($p_{\text{wt ctr vs Snx33 -/- ctr}}$ =2.542e-07, Mann-Whitney-U-Test, two-sided; $p_{\text{wt ctr vs wt low}}$ = 0.1341, Mann-Whitney-U-Test, two-sided; $p_{\text{Snx33 -/- ctr vs Snx33 -/- low}}$= 0.02264, Mann-Whitney-U-Test, two-sided; $p_{\text{wt low vs Snx33 -/- low}}$ =0.25 t = -1.1565, df = 108.88) and **i)** leading edge area. ($p_{\text{wt ctr vs Snx33 -/- ctr}}$ = 7.199e-05, Mann-Whitney-U-Test, two-sided; $p_{\text{wt ctr vs wt low}}$ = 0.6762, Mann-Whitney-U-Test, two-sided; $p_{\text{Snx33 -/- ctr vs Snx33 -/- low}}$ = 0.3537, Mann-Whitney-U-Test, two-sided; $p_{\text{wt low vs Snx33 -/- low}}$ = 0.0001791). n_{wt} = 82, $n_{\text{Snx33-/-}}$ = 102; Lower adhesion: n_{wt} = 53, $n_{\text{Snx33-/-}}$ = 72. **j)** Quantification of membrane ruffles area in wt and Snx33 -/- dHL-60 cells (p= 0.0001258, Mann-Whitney-U-Test, two-sided). N_{wt} = 175, $n_{\text{Snx33-/-}}$ = 170. Scale bars = 10 µm. p<0.001 (***), p<0.01 (**), p<0.05 (*). Box-plots: the lower and upper hinges correspond to the 25th and 75th percentile. The upper whisker extends from the hinge to the largest value, but no further than 1.5*IQR. The lower whisker extends from the hinge to the smallest value, but no lower than 1.5*IQR of the hinge. Data beyond the whiskers: black dots. Black line: median. Black dot: mean.

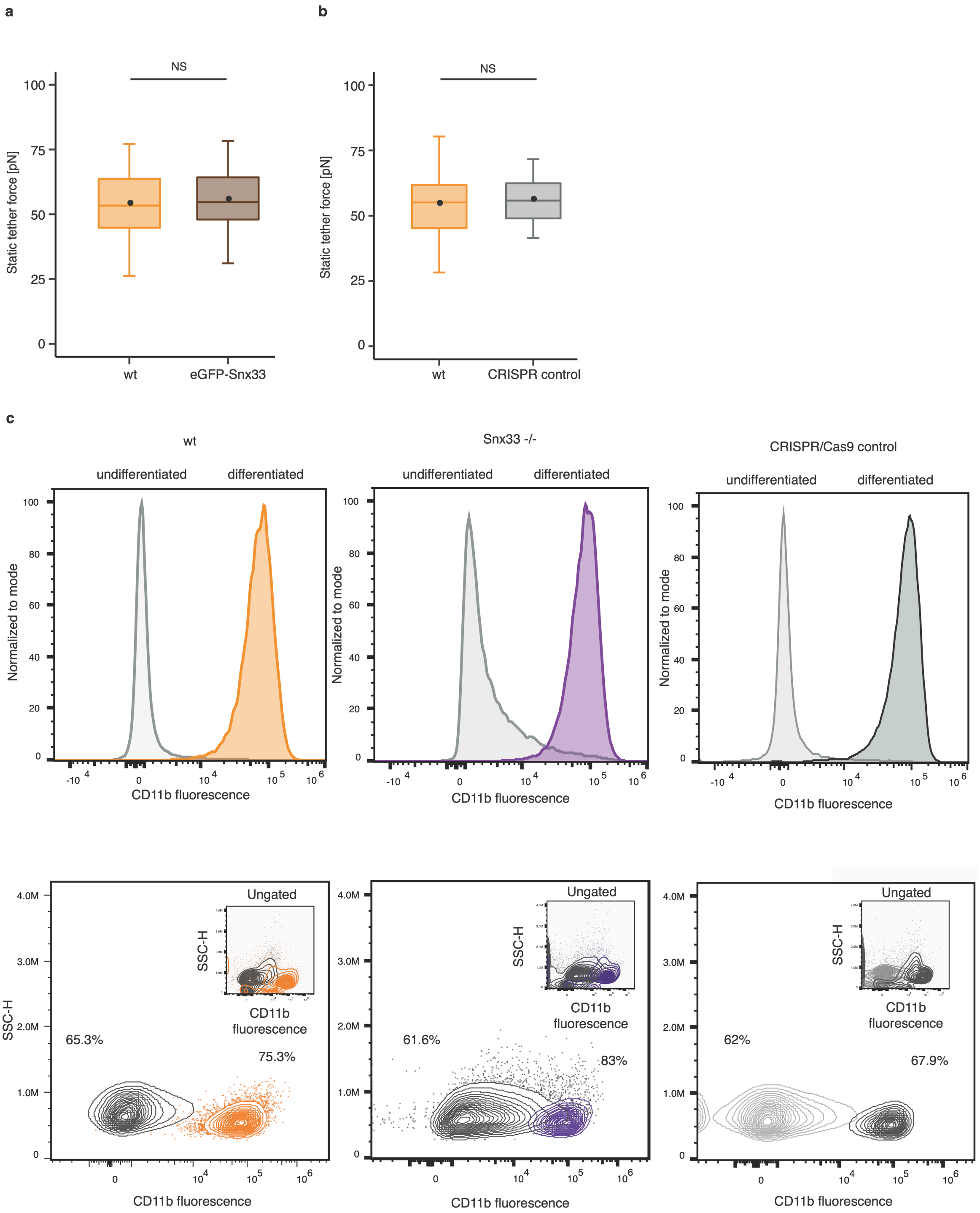

Supplementary Fig. 9: Snx33 -/- cell line characterization. a) Mean static tether force of wt (n = 42) and eGFP-Snx33 over-expressed (n = 42) dHL-60 cells from 6 independent experiments (p = 0.5255, t = -0.63759, df =80.889, two-sided). **b)** Mean static tether force of wt (n = 27) and CRISPR control (n = 28) dHL-60 cells from 4 independent experiments (p = 0.7783, t = 0.283, df = 52.793, two-sided). **c)** Representative histograms and contour plots of CD11b intensity of wild-type, Snx33 -/- and CRISPR/Cas9 control of HL-60 cells before and after 5 days of differentiation. Statistics: t-test and Mann-Whitney-U-test. $p<0.001$ (***), $p<0.01$ (**), $p<0.05$ (*). Box-plots: the lower and upper hinges correspond to the 25th and 75th percentile. The upper whisker extends from the hinge to the largest value, but no further than 1.5*IQR. The lower whisker extends from the hinge to the smallest value, but no lower than 1.5*IQR of the hinge. Data beyond the whiskers: black dots. Black line: median. Black dot: mean.

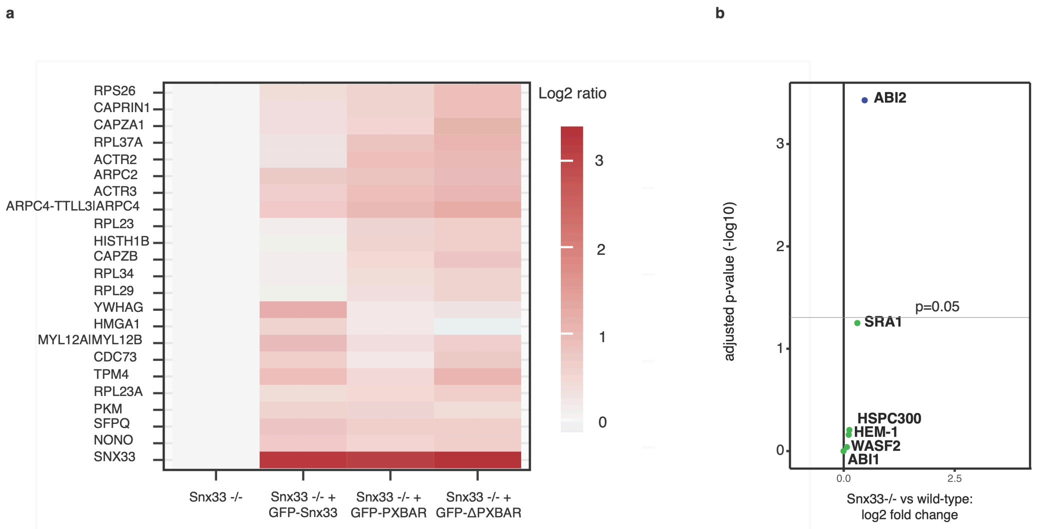

Supplementary Fig. 10: Snx33 interaction with Arp2/3 complex and WAVE2 expression. a) Heatmap showing the top 23 enriched proteins identified by mass spectrometry in coimmunoprecipitation of Snx33 and its truncations. **b)** Volcano plot showing differential RNA expression of WAVE2 complex components between wild-type and Snx33 -/- dHL60 cells (Wald test, two-sided).

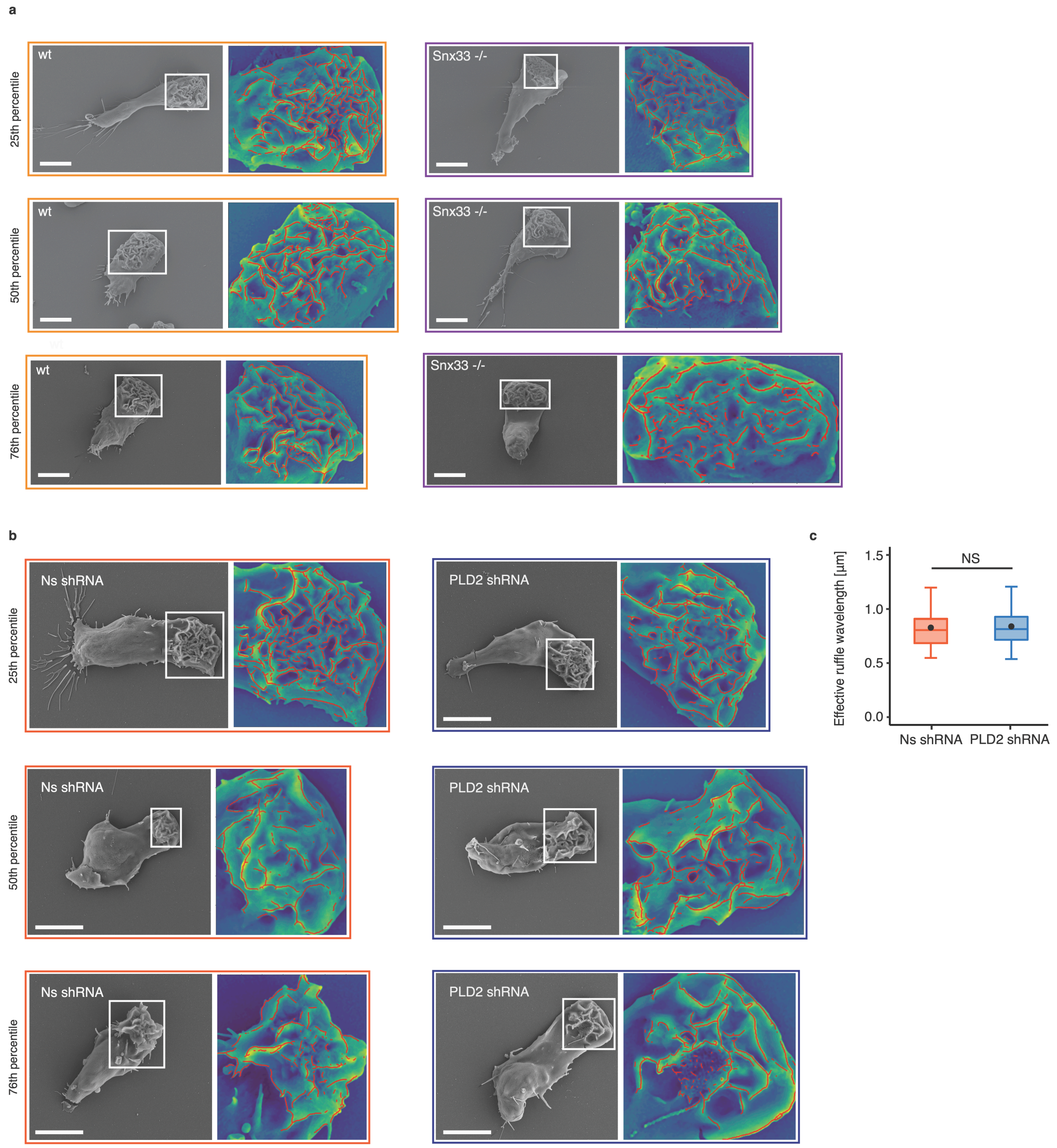

Supplementary Fig. 11: WAVE2 pattern size and ruffle wavelength increases upon knocking out Snx33. **a)** SEM images (25th, 50th and 76th percentile) with zoom-in of the overlay of the leading edge and ruffle segmentation (red) for wild-type and Snx33 -/- dHL-60 cells. n=3. **b)** SEM images (25th, 50th and 76th percentile) with zoom-in of the overlay of the leading edge and ruffle segmentation (red) for Nonsense and PLD2 KD dHL-60 cells. **c)** Quantification of effective ruffle wavelength in control (nonsense) and PLD2 KD dHL-60 cells ($p = 0.7304$, $t = 0.3449$, $df = 295.36$, two-sided). n = 149 (Nonsense), n = 153 (PLD2 KD). Scale bars = 10 µm. $p<0.001$ (***), $p<0.01$ (**), $p<0.05$ (*). Box-plots: the lower and upper hinges correspond to the 25th and 75th percentile. The upper whisker extends from the hinge to the largest value, but no further than 1.5*IQR. The lower whisker extends from the hinge to the smallest value, but no lower than 1.5*IQR of the hinge. Data beyond the whiskers: black dots. Black line: median. Black dot: mean.

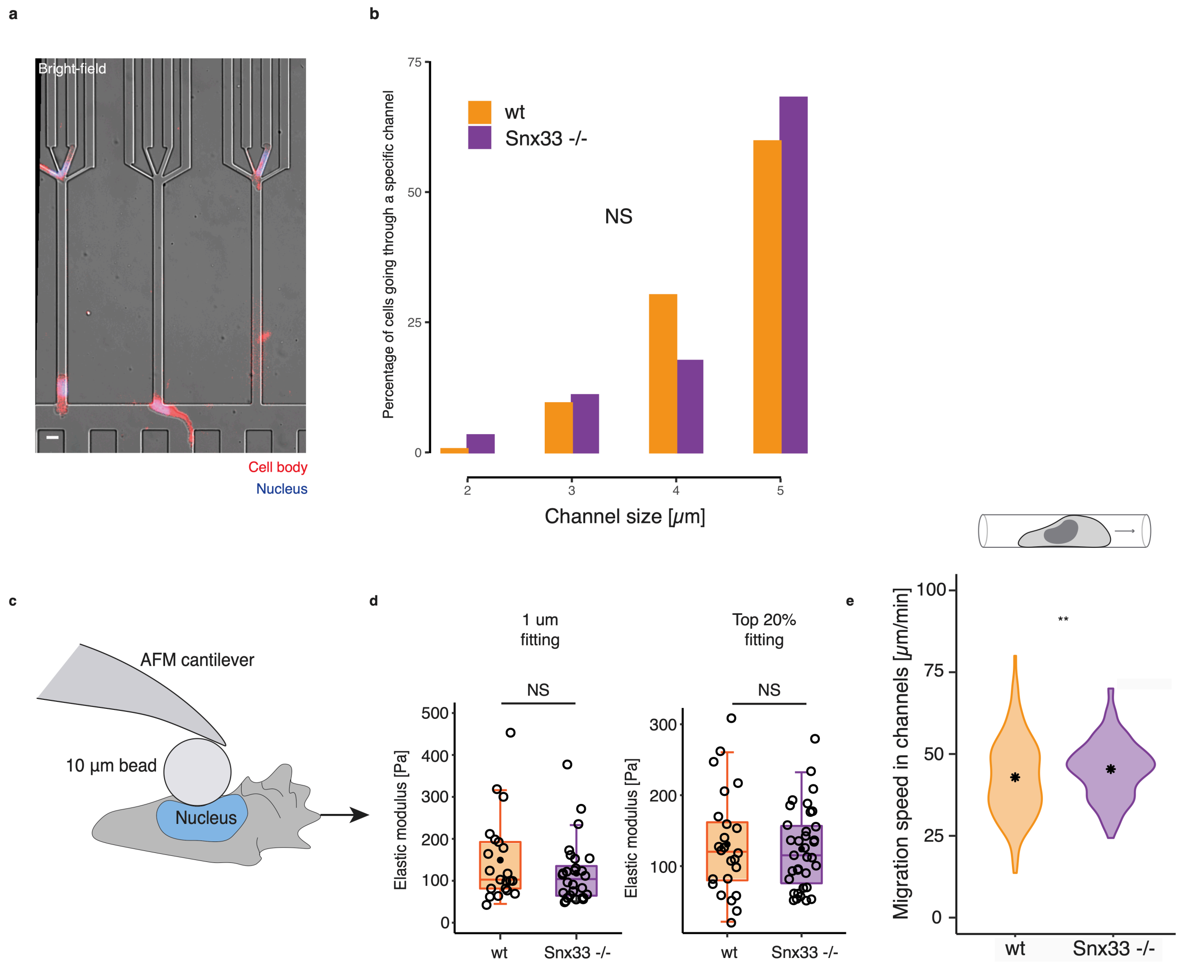

Supplementary Fig. 12: Snx33 knockout cells choose the path of least resistance independently of nuclear stiffness and migrate through channels faster than wild-type cells. a) Overlay of bright-field, nuclei and cell body images of cells migrating in PDMS-based devices with a decision point. **b)** Frequency of wt and Snx33 -/- cells choosing a channel of a certain size (p = 0.3909, Mann-Whitney-U-Test, two-sided). n = 159 for wt, n = 91 for Snx33 -/-. **c)** Schematic of an indentation experiment to measure nuclear stiffness. **d)** Quantification of nuclear stiffness of wt and Snx33 -/- dHL60 cells ($p_{1\mu m\ fitting}$ = 0.2397, Mann-Whitney-U-Test, two-sided ; p_{Top20} = 0.7122, t = 0.37142, df = 40.889, two-sided). n = 21 for wt, n = 28 for Snx33 -/-. Nuclear stiffness values were estimated by fitting 1 μm depth or Top 20% of indentation force curves using Herz model. **e)** Migration speed in straight channels (p = 0.008474, Mann-Whitney-U-Test, two-sided). n = 235 for wt, n = 169 for Snx33 -/-. Data from 3 independent biological replicates. Scale bar = 10 µm. p<0.001 (***), p<0.01 (**), p<0.05 (*). Box-plots: the lower and upper hinges correspond to the 25th and 75th percentile. The upper whisker extends from the hinge to the largest value, but no further than 1.5*IQR. The lower whisker extends from the hinge to the smallest value, but no lower than 1.5*IQR of the hinge. Data beyond the whiskers: black dots. Black line: median. Black dot: mean.

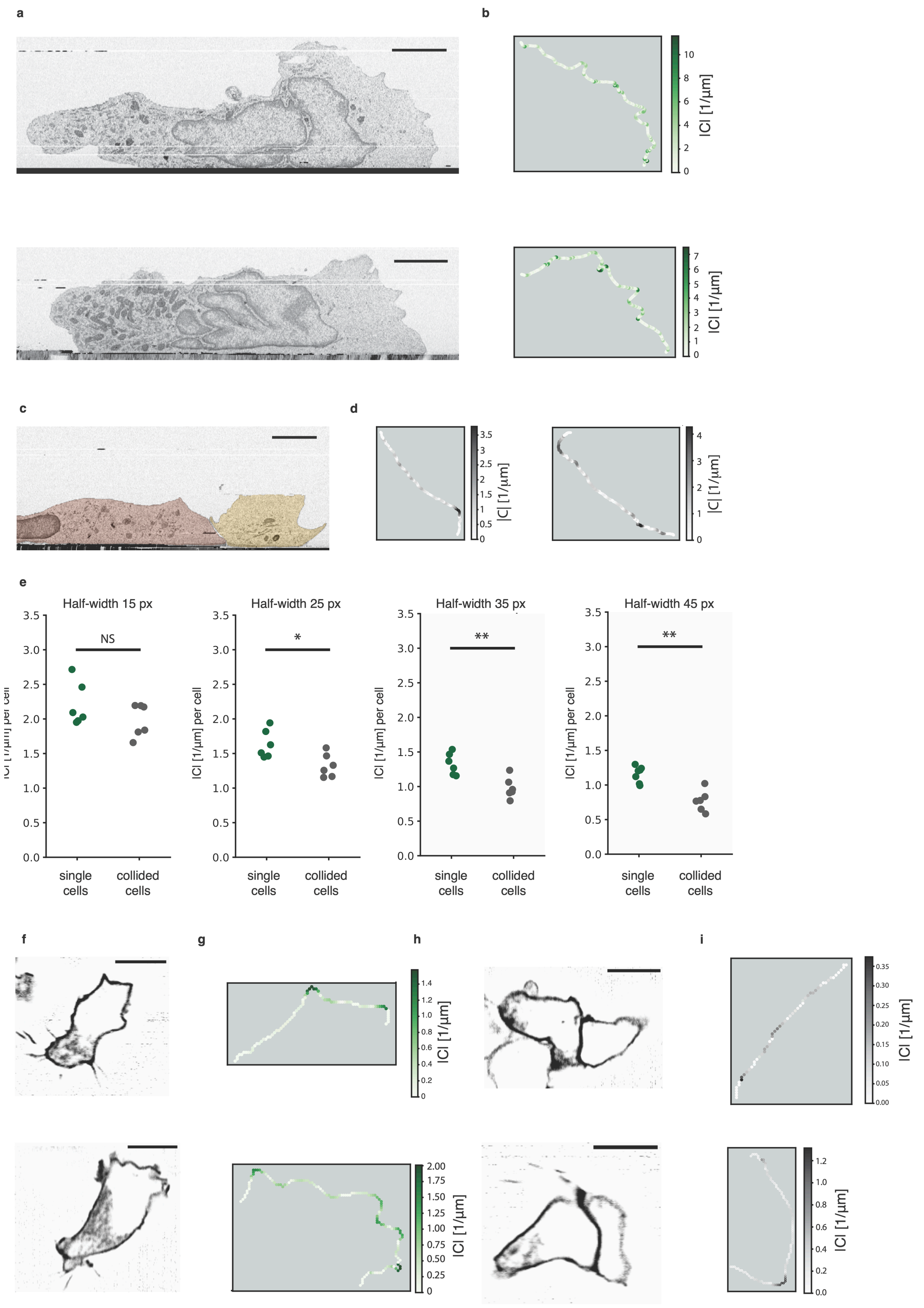

Supplementary Fig. 13: Cell collisions decrease average absolute curvature in the leading edge of migrating cells. a, c) Exemplary Cross-sections of single migrating (**a**) and colliding (**c**) dHL-60 cells by SBEM imaging. **b, d)** Visualization of the absolute curvature value in the leading edge (from **a** and **c**). **e)** Quantification of the absolute curvature value in the leading edge of single migrating cell at different pixel half-widths from SBEM data ($p_{HW15px} = 0.1914$, $t = 1.4086$, $df = 9.3212$; $p_{HW25px} = 0.01779$, $t = 2.85$, $df = 9.6788$; $p_{HW35px} = 0.002958$, $t = 3.9015$, $df = 9.9927$; $p_{HW45px} = 3.097e{-06}$, $t = 22.681$, $df = 5$, two-sided). **f, h)** Exemplary XY planes of single migrating (**f**) and colliding (**h**) dHL-60 cells, where a fluorescently labelled membrane (CAAX-mcherry) was imaged by lattice light sheet microscopy. **g, i)** Visualization of the absolute curvature at the leading edge (from **f** and **h**). Statistics: t-test or non-parametric Mann-Whitney-U-test. Scale bars = 10 µm. Scale bars = 10 µm. p<0.001 (***), p<0.01 (**), p<0.05 (*).

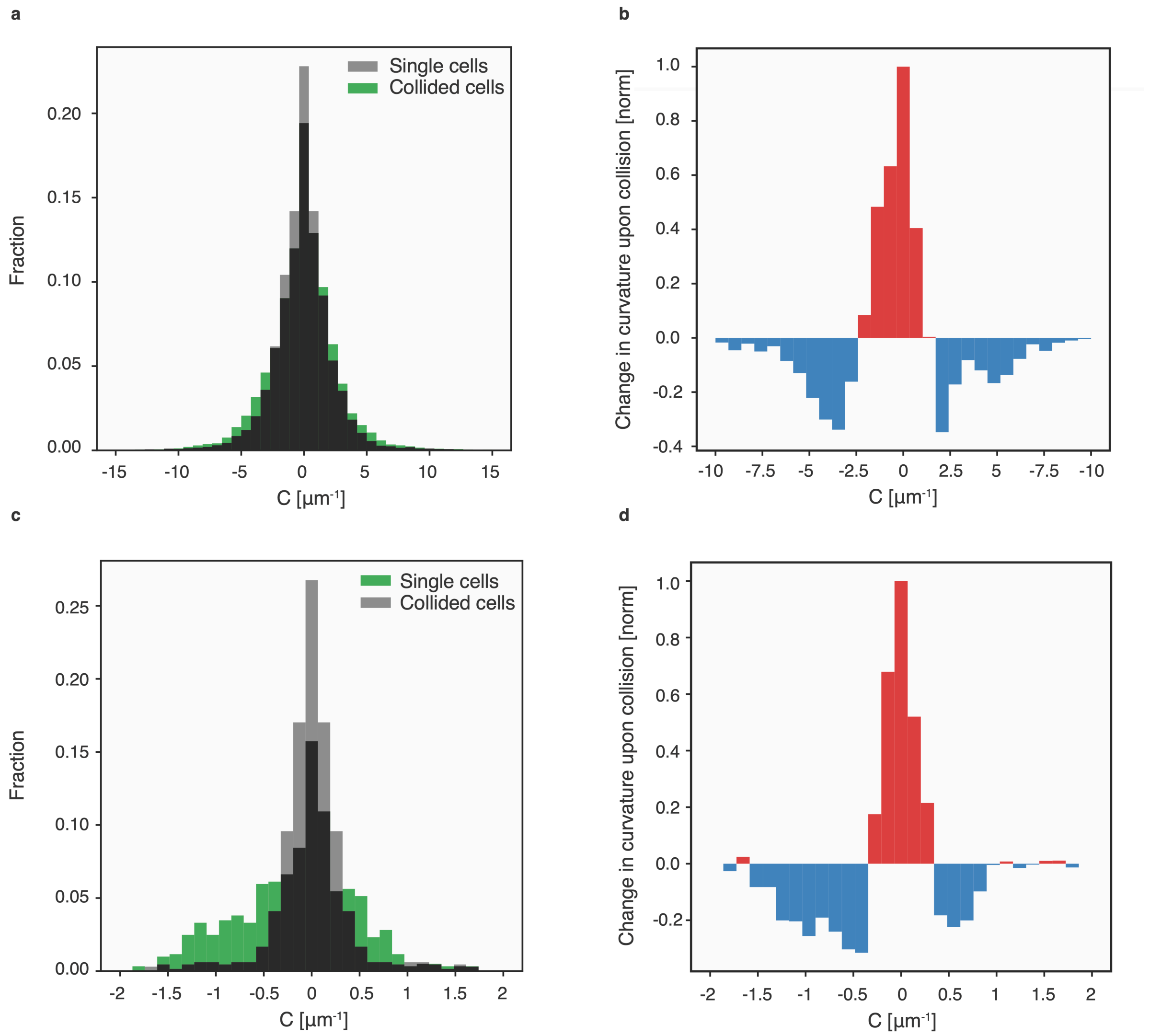

Supplementary Fig. 14: Quantification of positive and negative curvature and its change in single and collided migrating cells. a) Quantification of positive and negative curvature in the leading edge of single (n=6) and collided (n=6) leading edge of migrating cells (from images such as **Supplementary Fig. 13a, c**). Based on images acquired by SBEM imaging. Half-width = 20 pixels. **b)** Quantification of change in curvature distribution between collided (n=6) and single (n=6) cells. Based on images acquired by SBEM imaging. Half-width = 20 pixels. **c)** Quantification of positive and negative curvature in the leading edge of single (n=6) and collided (n=6) leading edge of migrating cells (from images such as **Supplementary Fig. 13f, h**). Based on images acquired by lattice light sheet microscopy. Half-width = 7 pixels. **d)** Quantification of change in curvature distribution between collided (n=6) and single (n=6) cells. Based on images acquired by lattice light sheet microscopy. 'C' denotes curvature. Half-width = 7 pixels.

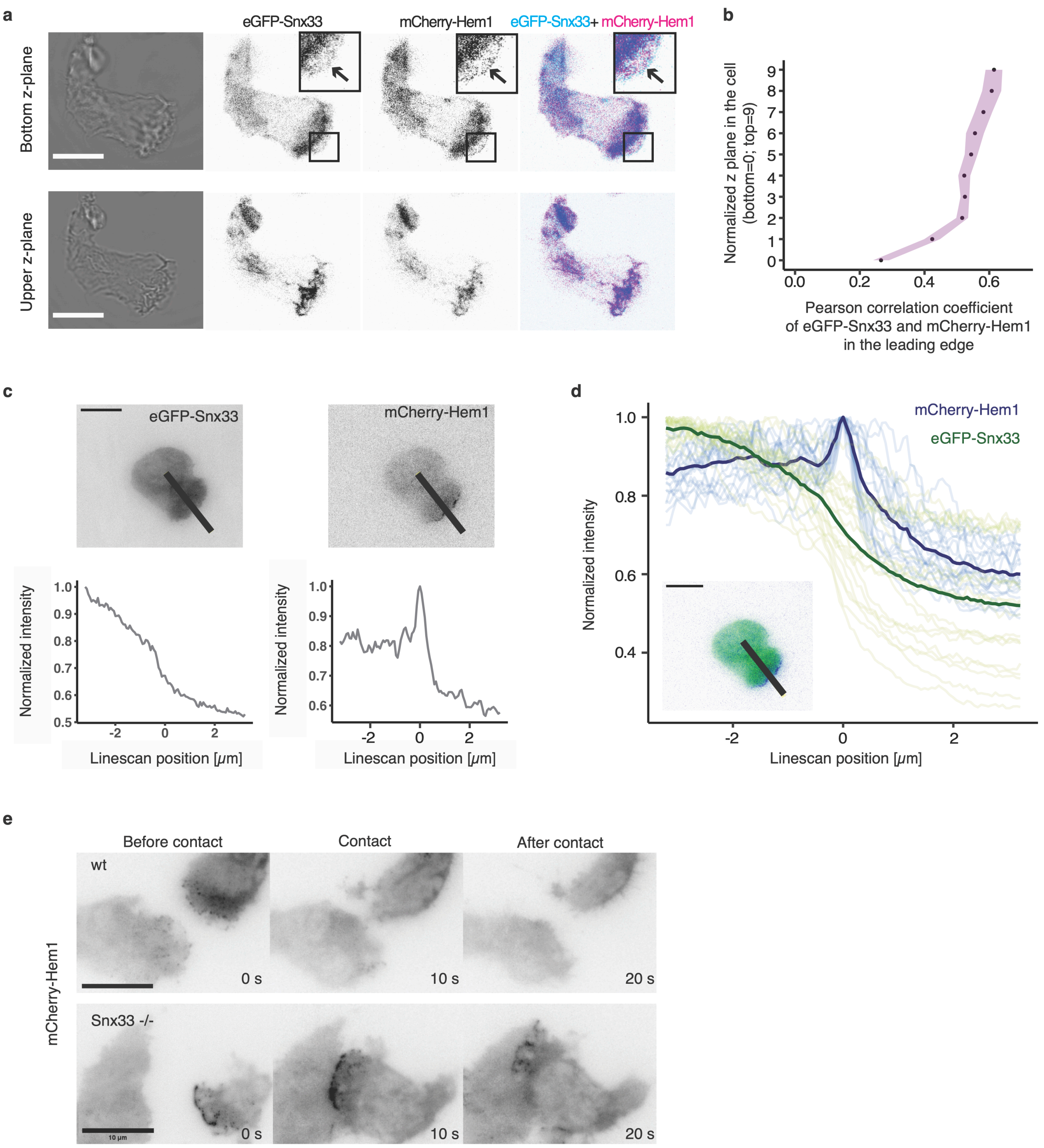

Supplementary Fig. 15: Snx33 is excluded from the protruding edge. a) Bright-field and confocal images of dHL-60 cell, eGFP-Snx33 and mCherry-Hem1 (component of WAVE2 complex) in the bottom (near-surface plane) and upper (close-to-the-cell top) z-planes. n=10 Arrows indicate the most protruding edge at the cell bottom. **b)** Pearson correlation of eGFP-Snx33 and mCherry-Hem1 in normalized z-planes in the leading edge. n = 10. Purple regions denote standard error of the mean. **c)** Exemplary images of fluorescently-tagged Snx33 and Hem1 and line scans through the leading edge in dHL-60 cells using TIRFM. n=22 **d)** Snx33 and Hem1 normalized intensity at the protrusion edge. **e)** TIRFM images of Hem1 signal before, during and after the contact event in wt and Snx33 -/- cells. Arrows are highlighting Hem-1 signal after contact event in wt and Snx33 -/- cells. wt (n = 9) and Snx33 -/- (n=10). Scale bar = 10 µm.

Table S1. Lipidomics from plasma membrane isolates from dHL60 cells.

Detected endogenous lipid classes	Mol%	Modelling of plasma membrane composition	Mol%
Cholesterol	39	Cholesterol	39
PC	22	PC	30
PC O-	8		
PE	3	PE	8
PE O-	5		
SM	13	SM	13
PS	6	PS	6
PI	4	PI	4

Supplementary Methods

Western blot

For immunodetection of Snx33 and GAPDH, $6x10^6$-$1.2x10^7$ HL-60 cells were lysed in RIPA Lysis and Extraction buffer (#89900, Thermo ScientificTM) according to manufacturer's instruction with supplementation of the protease inhibitors (#4693159001, Roche). Samples were denatured with 4xLaemmli buffer (#161- 0747, BioRad) containing 10% β-mercaptoethanol (#m6250, Sigma Aldrich) at 95° for 5 min. After SDS-PAGE and transfer, PVDF membrane with transferred proteins was blocked in 5% BSA in TBST and incubated over-night with 1:1000 dilution of anti-Snx33 (#orb331346, Biorbyt) or 1:80 000 dilution of anti-GAPDH (#NB300-221, Novus Biologicals). The blot was developed with secondary antibodies at 1:10 000 dilution of Donkey-Anti-Rabbit-HRP (711-035-152, Jackson Immuno Research) or Goat-Anti-Mouse-HRP (115-035-062, Jackson ImmunoResearch). For an example of presentation of full scan blots see Source Data file.

CD11b staining of HL-60 cells

After starvation, $1x10^5$ of undifferentiated and differentiated HL-60 cells were stained with 10 ul of Anti-Hu CD11b Alexa Fluor® 488 antibody solution (#A4-681-T100, Exbio). Fluorescence was measured on a Cytek® Aurora (Cytek) at the EMBL Flow Cytometry Core Facility and further analyzed and plotted using FlowJo (Version 10.9.0).

Nuclear stiffness measurements

For probing nuclear stiffness, 10 µm beads were glued to a Bruker cantilevers (MLCT-O10, C) and coated with 1% Pluronic solution using a CellHesion 200 AFM (Bruker), which is integrated into an Ecplipse Ti inverted light microscope (Nikon). Cantilevers were calibrated prior to bead gluing using the thermal noise method. Cell nuclei were identified based on nuclear staining and bright-field images. Cells were indented right on top of the nucleus. The data analysis was performed using the JPK Data Processing Software Version 6.1.183 in which indentation data were fitted 1 µm or top 20% with the Herz model to obtain estimates of nuclear stiffness, as previously reported[1].

Protein-Membrane interactions

The protein membrane interactions on the membrane surface were analyzed by considering the contacts between all protein beads and the phosphate (PO4) lipid beads in each frame. A residue was considered in contact with a lipid if the distance was below 8 Å for any lipid bead (**Supplementary Fig. 4e-h**). The time averaged percentage of contacts was mapped onto the BB beads (**Supplementary Fig. 4d**). The relative percentages of lipid contacts were determined from the total number of contacts in each frame (**Supplementary Fig. 4b**).

Protein insertion depth

The Fourier fit was applied to the PO4 beads of only the proximal membrane leaflet to which the PX-BAR domain binds to estimate the membrane surface. Then for each BB bead the XY position on the membrane plane was determined and whether it was above or below the fit plane. In each time step each residue was checked whether it was positioned above or below the fitted curve and the distance below the membrane was determined. The percentage of frames that a residue was below the fit was mapped to each BB residue (**Supplementary Fig. 3b**).

Plasma membrane lipidomics

Plasma membrane of HL-60 cells were isolated as previously[2] and analyzed by high-resolution shotgun lipidomics as performed in (PMID: 19174513; 21245337). In short, high-resolution Fourier Transform-mass spectrometry (FTMS) was carried out using an LTQ Orbitrap XL mass spectrometer (Thermo Fisher Scientific) equipped with a robotic nanoflow ion source, TriVersa NanoMate (Advion Biosciences). Lipid extracts were dissolved and diluted to yield an infusate composed of 7.5 mM ammonium formate in chloroform/methanol/2-propanol (1:2:4, vol./vol./vol.) for positive ion mode analysis and 0.01% methylamine in chloroform/methanol (1:5, vol./vol.) for negative ion mode analysis. Detected intact lipid ions were identified using ALEX123[3,4]. The molar abundances of lipids were determined by normalizing their peak intensities to that of spiked-in internal lipid standards and scaling by the molar spike amount of the internal standard. The levels of HL-60 plasma membrane lipid classes were furthermore cross-compared to the plasma membrane lipid compositions of Jurkat cells[5], MDCK cells[6] and BHK-21 cells[2].

Image analysis

For epifluorescence images (**Supplementary Fig. 8b-e, h, i**), single cells were manually selected using the ImageJ software. To segment the cell body, the contrast of bright-field images was enhanced using the equalize histogram function followed by a canny edge detection, a semi-manual closing of the obtained edges and filling the holes. To segment the leading edge, a similar strategy was used with semi-manual selection of the region with the enriched edges after the canny edge detection with higher values step. Based on the segmentation, a custom Python script was used for measurements.

For analysis of signal enrichment at the cell edge based on TIRFM images (**Supplementary Fig. 15d**), data on fluorescent intensity along the line of equal width and length were extracted from both channels of interest (eGFP-Snx33, mCherry-Hem1) using ImageJ. Data were aligned according to the highest fluorescence intensity of mCherry-Hem1 and normalized. Normalized mean intensity was calculated in both channels of interest for every point along the line scan and plotted using R.

For analysis of signal enrichment along protrusions based on TIRFM images (**Supplementary Fig. 6c**), data on fluorescent intensity along the line of equal width were extracted from both channels of interest (eGFP-Snx33 or eGFP-IRSp53, mCherry-CAAX) using ImageJ, taking into account only straight protrusions that connect to the cell body. Normalized mean intensity was calculated in both channels of interest for every point along the line scan and plotted using R.

Supplementary Discussion

At the plasma membrane, electrostatic interactions are the primary driver of protein recruitment and curvature sensing which is consistent with both the charge and the fact that the plasma membrane primarily consists of saturated lipids[7]. The Snx33 PX-BAR domain belongs to the class of BAR domains that primarily interact with the membrane via electrostatic interactions with negatively charged lipids. These interactions help targeting the protein to the plasma membrane. The Snx33 PX-BAR domain exhibits a large, positively charged basic surface (**Supplementary Fig. 2a-d**) which suggest preferential binding to negatively charged lipids.

Consistent with these considerations we found in our coarse-grained MD simulations that Snx33 PX-BAR resides on top of the membrane and primarily interacts with negatively charged lipids which are abundantly present in plasma membranes. Such observations have been previously made in experimental structural studies[8] and also in other simulation studies, for example for Amphiphysin N-BAR domains[9,10] and the PACSIN1 F-BAR domain[11]. In our simulations we found that Snx33 PX-BAR does not penetrate deeply into the membrane. Specifically, we fit the head group region (PO4 beads) of the membrane leaflet proximal to the Snx33 PX-BAR domain with a Fourier series to check the overall positioning of the Snx33 PX-BAR with respect to the membrane and its depth of insertion (**Supplementary Fig. 3**). We find that only some peripheral loops shallowly insert into the membranes (**Supplementary Fig. 3a**). The average maximal insertion depth of any residue in Snx33 is only around 4 Å with respect to the fit which is negligible considering the thickness of the membrane (**Supplementary Fig. 3b**). Therefore, tail saturation and properties of the hydrophobic core of the plasma membrane likely have little noticeable impact on the binding of the PX-BAR domain with the membrane. The interaction with the periphery of the headgroup region can be explained by the few hydrophobic residues and the large number of charges on the surface of Snx33.

Next, we checked the interactions of the Snx33 PX-BAR domain with the plasma membrane by analyzing the number of lipids and their types which are in contact within the protein (**Supplementary Fig. 4**). Hereby, all lipids within 6 Å were considered to be in contact. We found that a large amount of protein interactions are formed with POPI lipids. Even though only a small fraction of 4% of lipids are in the membrane composition, more than 20% of the interactions of PX-BAR with the membrane phospholipids are made up of interactions with POPI. Many of these POPI molecules have interactions with a duration of multiple microseconds. The amount of contacts with POPI is on par with interactions with the most abundant phospholipid POPC which is nearly 7.5 fold times more abundant in the membrane. The PX-BAR domain primarily binds these POPI molecules to the patches of lysine and arginine residues (**Supplementary Fig. 4c**). The interactions with POPC, DPSM and POPS are drastically shorter lived and not as specific. Much more exchange events can be observed for these lipid species. All these findings taken together are in line with the general understanding of plasma membrane binding via electrostatic interactions[7].

Supplementary References

1. Fischer, T., Hayn, A. & Mierke, C. T. Effect of Nuclear Stiffness on Cell Mechanics and Migration of Human Breast Cancer Cells. *Front Cell Dev Biol* **8,** 393 (2020).
2. Kalvodova, L. *et al.* The lipidomes of vesicular stomatitis virus, semliki forest virus, and the host plasma membrane analyzed by quantitative shotgun mass spectrometry. *Journal of Virology* **83,** 7996–8003 (2009).
3. Pauling, J. K. *et al.* Proposal for a common nomenclature for fragment ions in mass spectra of lipids. *PLoS ONE* **12,** e0188394 (2017).
4. Ellis, S. R. *et al.* Automated, parallel mass spectrometry imaging and structural identification of lipids. *Nat Meth* **15,** 515–518 (2018).
5. Zech, T. *et al.* Accumulation of raft lipids in T-cell plasma membrane domains engaged in TCR signalling. *EMBO J* **28,** 466–476 (2009).
6. Gerl, M. J. *et al.* Quantitative analysis of the lipidomes of the influenza virus envelope and MDCK cell apical membrane. *J Cell Biol* **196,** 213–221 (2012).
7. Bigay, J. & Antonny, B. Curvature, lipid packing, and electrostatics of membrane organelles: defining cellular territories in determining specificity. *Developmental Cell* **23,** 886–895 (2012).
8. Frost, A. *et al.* Structural Basis of Membrane Invagination by F-BAR Domains. *Cell* **132,** 807–817 (2008).
9. Arkhipov, A., Yin, Y. & Schulten, K. Four-scale description of membrane sculpting by BAR domains. *Biophysical Journal* **95,** 2806–2821 (2008).
10. Yin, Y., Arkhipov, A. & Schulten, K. Simulations of membrane tubulation by lattices of amphiphysin N-BAR domains. *Structure* **17,** 882–892 (2009).
11. Mahmood, M. I., Noguchi, H. & Okazaki, K.-I. Curvature induction and sensing of the F-BAR protein Pacsin1 on lipid membranes via molecular dynamics simulations. *Scientific Reports* **9,** 14557–11 (2019).
