# Supplementary material for: Sensing their plasma membrane curvature allows migrating cells to circumvent obstacles
Source: Nat Commun. 2023 Sep 13;14:5644. doi: 10.1038/s41467-023-41173-1 (PMC10499897; doi:10.1038/s41467-023-41173-1)
Supplement: Supplementary file 2 — Description of Additional Supplementary files [file 41467_2023_41173_MOESM2_ESM.pdf]

Description of Additional Supplementary files

Supplementary Video 1
Migrating HL-60 cell imaged by pTIRFM. Time lapse pTIRFM imaging of a dHL-60 cell stained with DiI using p-polarization (upper) and s-polarization (lower). Scale bar = 10 µm.

Supplementary Video 2
Migrating HL-60 cell with fluorescently labelled Snx33 (green) and membrane (magenta) imaged by lattice light sheet microscopy. Snx33 is enriched at the leading edge and localizes to membrane ruffles in migrating HL-60 cells. Scale bar = 10 µm.

Supplementary Video 3
Migrating HL-60 cell with fluorescently labelled IRSp53 and the accompanying bright-field image. IRSp53 is enriched at structures with negative (outward) curvature - retraction fibers and cell front. Scale bar = 10 µm.

Supplementary Video 4
TIRFM imaging of membrane (mCherry-CAAX) and leading edge segmentation in a wild-type HL60 cell. Scale bar = 10 µm.

Supplementary Video 5
TIRFM imaging of membrane (mCherry-CAAX) and leading edge segmentation in a Snx33 -/- HL60 cell. Scale bar = 10 µm.

Supplementary Video 6
TIRFM imaging of WAVE2 complex (eGFP-Hem1) its segmentation in a wild-type HL60 cell. Scale bar = 10 µm.

Supplementary Video 7
TIRFM imaging of WAVE2 complex (eGFP-Hem1) its segmentation in a Snx33 -/- HL60 cell. Scale bar = 10 µm.

Supplementary Video 8 Overlay of bright-field (grey) and nuclei (white) of wt and Snx33 -/- HL-60 cells migrating in PDMSbased devices with a decision point. Scale bar = 10 µm.

Supplementary Video 9 Overlay of bright-field (grey) and nuclei (blue) of wt and Snx33 -/- HL-60 cells migrating in PDMS-based devices with long channels and a single constriction. Scale bar = 10 µm.

Supplementary Video 10 TIRFM imaging of WAVE2 (eGFP-Hem1) and bright field of colliding wild-type HL-60 cells. Arrow indicates the first moment of cells contact. Scale bar = 10 µm.
