# Supplementary material for: Sensing their plasma membrane curvature allows migrating cells to circumvent obstacles
Source: Nat Commun. 2023 Sep 13;14:5644. doi: 10.1038/s41467-023-41173-1 (PMC10499897; doi:10.1038/s41467-023-41173-1)
Supplement: Supplementary file 13 — Reporting Summary [file 41467_2023_41173_MOESM13_ESM.pdf]

nature portfolio

Corresponding author(s): Alba Diz-Muñoz

Last updated by author(s): 20/7/23

Reporting Summary

Nature Portfolio wishes to improve the reproducibility of the work that we publish. This form provides structure for consistency and transparency in reporting. For further information on Nature Portfolio policies, see our Editorial Policies and the Editorial Policy Checklist.

Statistics

For all statistical analyses, confirm that the following items are present in the figure legend, table legend, main text, or Methods section.

n/a	Confirmed	
☐	☒	The exact sample size (*n*) for each experimental group/condition, given as a discrete number and unit of measurement
☐	☒	A statement on whether measurements were taken from distinct samples or whether the same sample was measured repeatedly
☐	☒	The statistical test(s) used AND whether they are one- or two-sided<br>*Only common tests should be described solely by name; describe more complex techniques in the Methods section.*
☒	☐	A description of all covariates tested
☐	☒	A description of any assumptions or corrections, such as tests of normality and adjustment for multiple comparisons
☐	☒	A full description of the statistical parameters including central tendency (e.g. means) or other basic estimates (e.g. regression coefficient) AND variation (e.g. standard deviation) or associated estimates of uncertainty (e.g. confidence intervals)
☐	☒	For null hypothesis testing, the test statistic (e.g. *F*, *t*, *r*) with confidence intervals, effect sizes, degrees of freedom and *P* value noted<br>*Give P values as exact values whenever suitable.*
☒	☐	For Bayesian analysis, information on the choice of priors and Markov chain Monte Carlo settings
☒	☐	For hierarchical and complex designs, identification of the appropriate level for tests and full reporting of outcomes
☐	☒	Estimates of effect sizes (e.g. Cohen's *d*, Pearson's *r*), indicating how they were calculated

Our web collection on statistics for biologists contains articles on many of the points above.

Software and code

Policy information about availability of computer code

Data collection	JPK SPM Software 6.1.183; SBEMImage development version (2021.08.dev); SmartSEM version 6.06 with Service Pack 4; DigitalMicrograph Version 3.51.3720.0
Data analysis	Numpy 1.21.5; Skimage 0.19.2; Mahotas 1.4.13; Matplotlib 3.5.2; Scipy 1.9.1; Shapely 1.8.4; Python 3.9.13; MDAnalysis 1.1.1; Python 3.8; GROMACS 2021.4; Python 3.6; JPK DP Software 6.1.183; FlowJo software (Version 10.9.0); Mascot (v2.2.07); Ilastik software Version 1.3.1b1 and 1.3.3post3; R version 3.2.1; DigitalMicrograph Version 3.51.3720.0; Pymol 2.4.2; o MDAnalysis 2.0; VMD 1.9.4; Python 3.8; bctpy v0.5.0; certifi v2019.9.11; cycler v0.10.0; decorator v4.4.1; glob2 v0.6; h5py v2.9.0; imageio v2.6.1; kiwisolver v1.0.1; matplotlib v3.0.3; mkl-fft v1.0.6; mkl-random v1.0.1; networkx v2.4; numpy v1.15.4; ordered-set v4.0.2; pandas v0.24.2; patsy v0.5.1; Pillow v6.2.1; PyLaTeX v1.4.1; pyparsing v2.4.0; python-dateutil v2.8.0; pytz v2019.1; PyWavelets v1.1.1; scikit-image v0.16.2; scikit-learn v0.20.3; scipy v1.1.0; seaborn v0.9.0; SimpleITK v1.1.0.dev354+g1b889; six v1.12.0; sklearn v0.0; statsmodels v0.9.0; tornado v6.0.2; xlrd v1.2.0<br><br>The source code for MD simulations and image analysis are available through figshare (10.6084/m9.figshare.22109204: https://figshare.com/articles/dataset/Supporting_data_for_Sensing_their_plasma_membrane_curvature_allows_migrating_cells_to_circumvent_obstacles_by_Ewa_Sitarska_Silvia_Dias_Almeida_Marianne_Sandvold_Beckwith_Julian_Stopp_Jakub_Czuchnowski_Marc_Siggel_Rita_Roessner_Aline_Tschanz/22109204) and on github (https://github.com/JakubCzuchnowski/Sensing-their-plasma-membrane-curvature-allows-migrating-cells-to-circumvent-obstacles.git).

For manuscripts utilizing custom algorithms or software that are central to the research but not yet described in published literature, software must be made available to editors and reviewers. We strongly encourage code deposition in a community repository (e.g. GitHub). See the Nature Portfolio guidelines for submitting code & software for further information.

Data

Policy information about availability of data

All manuscripts must include a data availability statement. This statement should provide the following information, where applicable:

- Accession codes, unique identifiers, or web links for publicly available datasets
- A description of any restrictions on data availability
- For clinical datasets or third party data, please ensure that the statement adheres to our policy

The RCSB PDB database was used in the study with accession number 4AKV. The RNAseq data have been deposited to the ArrayExpress collections from BioStudies with the accession number E-MTAB-12436. The mass spectrometry proteomics data have been deposited to the ProteomeXchange Consortium via the PRIDE74 partner repository with the dataset identifier PXD033666. The MD simulations data are available through figshare: 10.6084/m9.figshare.22109204. The raw numbers for charts and graphs are available in the Source Data file whenever possible. All other data and unique reagents that support this study are available from the corresponding authors upon request.

Human research participants

Policy information about studies involving human research participants and Sex and Gender in Research.

Reporting on sex and gender	n/a
Population characteristics	n/a
Recruitment	n/a
Ethics oversight	n/a

Note that full information on the approval of the study protocol must also be provided in the manuscript.

Field-specific reporting

Please select the one below that is the best fit for your research. If you are not sure, read the appropriate sections before making your selection.

☒ Life sciences ☐ Behavioural & social sciences ☐ Ecological, evolutionary & environmental sciences

For a reference copy of the document with all sections, see nature.com/documents/nr-reporting-summary-flat.pdf

Life sciences study design

All studies must disclose on these points even when the disclosure is negative.

Sample size	No statistical methods were used to determine necessary sample sizes, instead they were estimated based on recent studies using similar methodology that include the following:<br>Graziano, B. R. et al. Cell confinement reveals a branched-actin independent circuit for neutrophil polarity. PLoS Biol 17, e3000457–34 (2019).<br>Diz-Muñoz, A. et al. Membrane Tension Acts Through PLD2 and mTORC2 to Limit Actin Network Assembly During Neutrophil Migration. PLoS Biol 14, e1002474–30 (2016).<br>Renkawitz, J. et al. Nuclear positioning facilitates amoeboid migration along the path of least resistance. Nature 568, 546–550 (2019).<br>Leithner, A. et al. Diversified actin protrusions promote environmental exploration but are dispensable for locomotion of leukocytes. Nature Cell Biology 18, 1253–1259 (2016).<br>Pipathsouk, A. et al. The WAVE complex associates with sites of saddle membrane curvature. J Cell Biol 220, (2021).<br>Houk, A. R. et al. Membrane tension maintains cell polarity by confining signals to the leading edge during neutrophil migration. Cell 148, 175–188 (2012).
Data exclusions	No data was excluded post analysis. Some analysis pipelines used internal quality control checks to screen their raw inputs to assure only proper quality data was analyzed.
Replication	All experiments have been performed with at least 2 biological replicates and almost all had at least 3 biological replicates. In singular cases the 3rd replicate was not possible due to limitations in equipment, time availability or sample viability reasons. All attempts at replication were successful.
Randomization	Where applicable (experiments with multiple conditions such as in Fig. 1e; Fig. 2b-i; Fig. 3b, d, f; Fig. 4a-g, q; SF6; SF8b-j; SF9a-c, SF. 10; SF11c, SF12b, d, e) conditions were randomized by changing the order in which data was acquired/processed/analyze to remove possible sources of bias connected to timing. Randomization was not possible when one type of sample was analyzed (Fig. 1a-d, f-g;SF1), when the data was collected together and analyzed by an automated method (Fig. 1g, k-m; Fig. 4h-m; SF13, SF14) or when the method used was inherently randomized (Fig. 1h-j; SF2, SF3, SF4).

Blinding	For semi-automatic image analysis requiring human input researchers were blinded by randomizing images and encoding filenames to remove all possible insight into which condition was being analyzed. Thus, investigators who collected and analyzed data were blinded to group allocation. For automatic analysis blinding was not required, so investigators were not blinded to group allocation. For experiments in which one type of data was analyzed blinding was not required.

Reporting for specific materials, systems and methods

We require information from authors about some types of materials, experimental systems and methods used in many studies. Here, indicate whether each material, system or method listed is relevant to your study. If you are not sure if a list item applies to your research, read the appropriate section before selecting a response.

Materials & experimental systems

n/a	Involved in the study
☐	☒ Antibodies
☐	☒ Eukaryotic cell lines
☒	☐ Palaeontology and archaeology
☒	☐ Animals and other organisms
☒	☐ Clinical data
☒	☐ Dual use research of concern

Methods

n/a	Involved in the study
☒	☐ ChIP-seq
☐	☒ Flow cytometry
☒	☐ MRI-based neuroimaging

Antibodies

Antibodies used	anti-Snx33 (#orb331346, Biorbyt); anti-GAPDH (#NB300-221, Novus Biologicals); Donkey-Anti-Rabbit-HRP (711-035-152, Jackson ImmunoResearch); Goat-Anti-Mouse-HRP (115-035-062, Jackson ImmunoResearch); CD11b Alexa Fluor® 488 antibody solution (#A4-681-T100, Exbio)
Validation	All used antibodies were acquired commercially and validated by their producers.<br>anti-Snx33 (#orb331346)) - is a polyclonal antibody; The immunogen is a synthetic peptide directed towards the C-terminal region of Human SNX33; validated by Western blot analysis of human 721_B tissue by Biorbyt. Reactivity: human.<br>anti-GAPDH (#NB300-221) - This GAPDH antibody was developed against full length purified porcine GAPDH; Reactivity: Hu, Mu, Rt, Po, Av, Bv, Ca, Ch, ChHa, Dr, Eq, Fe, Ha, Ma-Op, Pm, Rb, Sh, Ze; Monoclonal. Validated by customer in human nucleus pulposus cell culture (40ug/lane), Validated by publication in 15 confirmed species: Human, Mouse, Rat, Bovine, Canine, Chicken, Chinese Hamster, Drosophila, Opossum, Porcine, Primate, Rabbit, Sheep, WB, Zebrafish and in 8 applications: Chemotaxis, ICC/IF, IF/IHC, IHC-Fr, IP, PCR, Single Cell Western, WB.

Eukaryotic cell lines

Policy information about cell lines and Sex and Gender in Research

Cell line source(s)	HL60 cells were bought from ATCC and a single cell clone was isolated to optimize differentiation and growth. In the current study that cell line was used as starting material to generate the Snx33KO one as well as all fluorescently labelled cell lines. Cell line with GFP-tagged IRSp53 was a kind gift from Orion Weiner (from: Pipathsouk, A. et al. The WAVE complex associates with sites of saddle membrane curvature. J Cell Biol 220, (2021).).
Authentication	The original HL60 cell line and Snx33 KO cell line were authenticated in the study by gDNA sequencing and Western Blot (anti-Snx33 (#orb331346, Biorbyt)), respectively.
Mycoplasma contamination	All cell lines tested negative for Mycoplasma contamination.
Commonly misidentified lines<br>(See ICLAC register)	No commonly misidentified cell lines were used in the study.

Flow Cytometry

Plots

Confirm that:

☒ The axis labels state the marker and fluorochrome used (e.g. CD4-FITC).

☒ The axis scales are clearly visible. Include numbers along axes only for bottom left plot of group (a 'group' is an analysis of identical markers).

☒ All plots are contour plots with outliers or pseudocolor plots.

☒ A numerical value for number of cells or percentage (with statistics) is provided.

Methodology

Sample preparation	Sample preparation for staining of CD11b in HL60 cells: After starvation, 1x10^5 of undifferentiated and differentiated HL-60 cells were stained with 10 ul of Anti-Hu CD11b Alexa Fluor® 488 antibody solution (#A4-681-T100, Exbio).
Instrument	Cytek® Aurora (Cytek)
Software	FlowJo
Cell population abundance	Cell population abundance in reported experiments was high as the input sample originated from cell culture, thus only debris and cell doublets were removed from analysis via gating and sample purity evaluation was not required.
Gating strategy	Simple gating strategies were used as the sample came from a pure culture. Debris and cell doubles were removed by their FSC/SSC values and live cells were selected based on a viability staining.

☒ Tick this box to confirm that a figure exemplifying the gating strategy is provided in the Supplementary Information.
